# Mechanisms of Isothiocyanate Detoxification in Larvae of Two Belowground Herbivores, *Delia radicum* and *D. floralis* (Diptera: Anthomyiidae)

**DOI:** 10.3389/fphys.2022.874527

**Published:** 2022-04-29

**Authors:** Rebekka Sontowski, Cervin Guyomar, Yvonne Poeschl, Alexander Weinhold, Nicole M. van Dam, Daniel G. Vassão

**Affiliations:** ^1^ Molecular Interaction Ecology, German Centre for Integrative Biodiversity Research (iDiv) Halle-Jena-Leipzig, Leipzig, Germany; ^2^ Institute of Biodiversity, Friedrich Schiller University, Jena, Germany; ^3^ GenPhySE, Université de Toulouse, INRAE, ENVT, Castanet Tolosan, France; ^4^ Bioinformatics Unit, German Centre for Integrative Biodiversity Research (iDiv) Halle-Jena-Leipzig, Leipzig, Germany; ^5^ Institute of Computer Science, Martin Luther University Halle-Wittenberg, Halle, Germany; ^6^ Max Planck Institute for Chemical Ecology, Jena, Germany

**Keywords:** plant-insect interaction, detoxification, allelochemicals, herbivory, pest, metabolism, glucosinolates, Brassicaceae

## Abstract

Like aboveground herbivores, belowground herbivores are confronted with multiple plant defense mechanisms including complex chemical cocktails in plant tissue. Roots and shoots of Brassicaceae plants contain the two-component glucosinolate (GSL)-myrosinase defense system. Upon cell damage, for example by herbivore feeding, toxic and pungent isothiocyanates (ITCs) can be formed. Several aboveground-feeding herbivores have developed biochemical adaptation strategies to overcome the GSL-ITC defenses of their host plant. Whether belowground herbivores feeding on *Brassica* roots possess similar mechanisms has received little attention. Here, we analyze how two related belowground specialist herbivores detoxify the GSL-ITC defenses of their host plants. The larvae of the fly species *Delia radicum* and *D. floralis* are common pests and specialized herbivores on the roots of Brassicaceae. We used chemical analyses (HPLC-MS/MS and HPLC-UV) to examine how the GSL-ITC defense system is metabolized by these congeneric larvae. In addition, we screened for candidate genes involved in the detoxification process using RNAseq and qPCR. The chemical analyses yielded glutathione conjugates and amines. This indicates that both species detoxify ITCs using potentially the general mercapturic acid pathway, which is also found in aboveground herbivores, and an ITC-specific hydrolytic pathway previously characterized in microbes. Performance assays confirmed that ITCs negatively affect the survival of both species, in spite of their known specialization to ITC-producing plants and tissues, whereas ITC breakdown products are less toxic. Interestingly, the RNAseq analyses showed that the two congeneric species activate different sets of genes upon ITC exposure, which was supported by qPCR data. Based on our findings, we conclude that these specialist larvae use combinations of general and compound-specific detoxification mechanisms with differing efficacies and substrate preferences. This indicates that combining detoxification mechanisms can be an evolutionarily successful strategy to handle plant defenses in herbivores.

## 1 Introduction

Feeding on plants is a great challenge, as plants have evolved a large diversity of morphological and chemical defense strategies. Most plants produce complex chemical cocktails to prevent or reduce feeding damage (Büchel et al., 2016; Lackus et al., 2018). These compounds can act as repellents through their bitter taste and pungent aromas, be poisonous, or reduce the digestibility of plant tissues (Wittstock and Gershenzon 2002; Biere et al., 2004; Rehman et al., 2012). Plants of the Brassicaceae family defend themselves chemically with a two-component system consisting of glucosinolates (GSLs) and the myrosinase (β-thioglucosidase) enzyme family. GSLs themselves are non-toxic to herbivores. However, once hydrolyzed by myrosinases, for example upon attack by a herbivore, they form an unstable aglycone that rearranges to form toxic isothiocyanates (ITCs), next to other biologically active compounds such as nitriles (Wittstock et al., 2003).

Nevertheless, many herbivores have adapted to chemical defenses and can feed with near impunity on well-defended plant tissues. Mirroring the great diversity of plant defensive compounds, herbivorous adaptation mechanisms are also highly diverse, but how these strategies contribute to the success and survival of herbivores are often not well understood (Wittstock and Gershenzon 2002; Heidel-Fischer and Vogel 2015). To adapt to the GSL-myrosinase defense system, insect herbivores have evolved different mechanisms. These include, for instance, the prevention of plant cell disruption, modification of the substrate (GSL) to generate non-hydrolyzable derivatives, or the diversion of the enzymatic conversion towards less toxic hydrolysis products (Ratzka et al., 2002; Wittstock et al., 2004; Kim et al., 2008; Malka et al., 2020). Most herbivores, including generalist caterpillars with a broad host plant range, possess mechanisms to detoxify the hydrolysis products (ITCs). Two ITC detoxification pathways have been described so far. Several insects use the mercapturic acid pathway, which is a general detoxification pathway employing glutathione-*S*-transferase (GST) activities to metabolize ITCs into non-toxic glutathione conjugates and derivatives that can be excreted with the feces (Yu 1982; Schramm et al., 2012). The second pathway is the hydrolysis of ITCs to form amines followed by further metabolism into acetamides (Beran et al., 2018). These products are less toxic and more easily excretable. This pathway has been found in insects and microbes (Beran et al., 2018; Chen et al., 2020; Fan et al., 2011). Commonly, the expression of genes coding for detoxification enzymes, e.g. specific glutathione-*S*-transferases or P450s, is upregulated only upon exposure to ITCs (Halon et al., 2015). It has been hypothesized that the specific upregulation of enzyme production reduces the energetic costs of detoxification (Fürstenberg-Hägg et al., 2013).

So far, all of the known GSL/ITC detoxification mechanisms have been described in aboveground herbivores. Even though belowground herbivores feeding on *Brassica* roots are exposed to similar or even higher levels of GSLs and ITCs (Crespo et al., 2012; Tsunoda et al., 2017), it is largely unknown which biochemical adaptations they possess towards the GSL-ITC system. Therefore, we examined the biochemical mechanisms of GSL and ITC metabolism in two closely related belowground herbivores, the larvae of the cabbage root fly (*Delia radicum*, Linné, Diptera: Anthomyiidae) and the turnip root fly (*D. floralis*, Fallén). The adults live aboveground where they consume pollen and nectar. Females oviposit on the soil near the root-shoot interface of brassicaceous plants. After hatching, the neonate larvae crawl into the soil, where they feed on the roots (Birch et al., 1992; Klingen et al., 2000). Considering that several crops, such as cabbages, radishes, and rapeseed belong to the Brassicaceae, the larvae of the two fly species are notorious agricultural pests, causing millions of dollars of crop losses annually in Europe and Northern America (Wang et al., 2016).

Both species share many biological and behavioral traits. Besides being close relatives, having a similar morphology, feeding mode and host plant range, they also overlap in their geographic distribution, namely the northern hemisphere (Coaker and Finch 1970; Darvas and Szappanos 2003). Therefore, we hypothesized that *D. radicum* and *D. floralis* larvae handle the GSL-ITC defense system similarly. Because herbivores may interfere with the GSL-ITC system at multiple points, we first studied at which position of the activation pathway (GSL substrates or ITC hydrolysis products) the larvae might divert or modify these chemical defenses. To test this, we first examined whether *D. radicum* and *D. floralis* larvae catabolize GSLs or ITCs. We incubated larval gut extracts with different GSLs or ITCs, after which we analyzed the breakdown products by liquid chromatography with tandem mass spectrometry (LC-MS/MS) and high-performance liquid chromatography-ultraviolet (HPLC-UV). We focused mostly on 2-phenylethyl glucosinolate (2PE-GSL) and 2-phenylethyl isothiocyanate (2PE-ITC), as these are the most common GSLs and ITCs in *Brassica* roots (van Dam et al., 2009; van Dam et al., 2012). We also tested how the aliphatic GSL 4-(methylsulfinyl)butyl glucosinolate (4MSOB-GSL) and its corresponding ITC (4-(methylsulfinyl)butyl isothiocyanate, 4MSOB-ITC) was catabolized. This is the predominant GSL/ITC in the model plant *Arabidopsis thaliana* Col-0 and also occurs in the roots of several *Brassica* species (Bhandari et al., 2015). In addition, we identified candidate detoxification genes that may be involved in the detoxification of 2PE-ITC in the larvae of both species, using transcriptomic data. We thereby used an assembled and annotated genome of *D. radicum* as the reference genome (Sontowski et al., 2022). After selecting these candidate genes, we used qPCR analyses to study their response to different levels of 2PE- and 4MSOB-ITCs. Finally, we examined the effects of 2PE- and 4MSOB-ITCs and the larval breakdown products on *D. radicum* and *D. floralis* development using a performance assay. By combining these different approaches, we could assess similarities and differences in the underlying mechanisms as well as the biological effects of ITC detoxification in both root herbivores.

## 2 Material and Methods

### 2.1 Insect Rearing

*D. radicum* and *D. floralis* samples derived from a laboratory culture at the German Centre for Integrative Biodiversity Research in Leipzig, Germany which was established 7 years ago. The *D. radicum* rearing started with collected pupae from a cabbage field in Brittany, France, kindly provided by Dr. Anne-Marie Cortesero (University of Rennes, France) and the *D. floralis* rearing with pupae from a laboratory culture which were kindly provided by Dr. Maria Björkman (Bioforsk–Norwegian Institute of Agricultural and Environmental Research, Norway). The colonies have been maintained under controlled environmental conditions as described in Sontowski et al. (2019). We collected eggs and larvae for all following experiments from these cultures and performed the experiments at constant temperature of 20°C, 16:8 h light:dark and relative humidity of 70 ± 5% in a Percival Reach-In chamber (CLF Plant climatic, Wertingen, Germany).

### 2.2 Glucosinolate Breakdown by Gut Extracts of *D. radicum* and *D. floralis* Larvae

In the first experiment, we tested whether gut extracts from *D. radicum* and *D. floralis* larvae, which include also their gut microbiome, contain enzymes to catabolize GSLs. Larvae were fed on turnip and at the end of the 2^nd^ larval developmental stage (instar), the larvae were frozen at −20°C for 45 min, surface-sterilized (2 min in 0.2% bleach, neutralized by 1 min in potassium thiosulfate and rinsed three times with 70% ethanol) and had their guts dissected. We pooled 10 guts per replicate and manually homogenized them in 100 µl autoclaved ddH_2_O using a pestle. To exclude effects of different protein concentrations in the extracted samples between the different species, we measured the protein concentration in a 1:500 dilution of the samples using the Micro BSA protein assay kit (Thermo Scientific, Rockford, IL, United States) according to the supplier’s recommendations. Protein concentration was measured on a Jasco V-630 spectrophotometer (Jasco, Groß-Umstadt, Germany), and determined from three replicates. Extracts prepared from both species contained the same range of protein concentrations. For this experiment, we used the following treatments: 1) gut extracts of *D. radicum* larvae or 2) *D. floralis* larvae incubated at room temperature. 3) gut extracts of *D. radicum* larvae or 4) *D. floralis* larvae heated for 7 min at 95°C to reduce the microbial activity and to denature proteins. Each treatment contained three biological replicates. To test whether GSLs were degraded by the larval gut extracts, we added 67 µl of 2PE-GSL solution (300 μg/ml in H_2_O, 2-phenylethyl glucosinolate, also called gluconasturtiin; class: benzenic GSL, Phytoplan, Heidelberg, Germany, >97.0% purity) and 70 µl 4MSOB-GSL solution (300 μg/ml in H_2_O, 4-(methylsulfinyl)butyl GSL, also called glucoraphanin; class: aliphatic GSL, Phytoplan, Heidelberg, Germany, >97.0% purity) to all samples and incubated them for 1 h at 25°C. In addition, we analyzed three replicates of 10 normal gut extracts treated as described previously, but without adding external GSLs to consider GSL residues from the food in the gut. All reactions were stopped by adding 85% methanol and boiling the samples for 5 min at 92°C. The GSLs were extracted and analyzed according to Grosser and van Dam (2017). Briefly, GSLs were desulphated and analyzed with reverse phase Ultra High Performance Liquid Chromatography (UHPLC) equipped with a photodiode array detector (PDA; Thermo Scientific Ultimate 3000 Series, Thermo Fisher Scientific, Waltham, MA, United States) at 229 nm. We injected 50 µl per sample. Desulphated GSLs were separated with a reverse-phase C_18_ column (4.6 × 150 mm, 3 μm, Thermo Fisher Scientific, Schwerte, Germany) connected with a C_18_ pre-column (10 × 4.6 mm, 5 μm particle size) using the parameters described in Grosser and van Dam (2017). After separation, the identification of desulphated GSLs was carried out based on retention time and UV spectra compared to commercially available reference standards (Phytoplan, Heidelberg, Germany). Desulphated GSLs were quantified using sinigrin as an external standard and response factors as described in Grosser and van Dam (2017). Data were processed using Thermo Scientific Chromeleon Chromatography Data System software vs 7.2 SR5 MUa (Thermo Fisher Scientific, Waltham, MA, United States).

### 2.3 Isothiocyanates Detoxification by Gut Extracts of *D. radicum* and *D. floralis* Larvae

To test whether ITCs were degraded by the larval gut extracts, which includes also their gut microbiome, we repeated the experiment above (GSL-breakdown experiment) regarding the preparation of larvae, treatments and replicates. Again, we pooled the extracted guts of 10 larvae and mashed them in 80 µl potassium phosphate buffer (0.1 M, pH 7). Before adding ITCs instead of GSLs, we added 10 µl ZnSO_4_ (0.001 M) to each sample. Thereafter, 10 µl of 4MSOB-ITC (1 mg/ml in ethanol, MCE, Sollentuna, Sweden, purity >98%) or 10 µl of 2PE-ITC solutions (0.5 mg/ml in DMSO, Sigma-Aldrich, St. Louis, Missouri, United States, purity 99%) were added to the samples (*n* = 3 per condition group and ITC). To detect possible 4MSOB-ITC or 2PE-ITC from the food residues in the extracted guts, three additional replicates of larval guts from both species were tested without adding external ITCs. These samples were treated as described previously but instead of ITC, only the corresponding solvents were added (10 µl of 96% ethanol or 10 µl of 100% DMSO). All reactions were incubated for 1 h at 25°C. To stop the reactions, we added 10 µl of glacial acetic acid and vortexed quickly. All samples were subsequently centrifuged for 10 min at 10°C at 12.000 rpm (adapted from Schramm et al. (2012) and Jeschke et al. (2016)). The supernatant was transferred to a new vial and stored at −20°C until measurement. An aliquot of 1 µl was injected on an Agilent 1260 series HPLC system (Agilent Technologies, Boeblingen, Germany) coupled with an API5000 tandem mass spectrometer (Applied Biosciences, Darmstadt, Germany) using the HPLC parameters described in Chen et al. (2020). Separation of compounds was performed using a Zorbax Eclipse XDB-C-18 column (50 × 4.6 mm, 1.8 μm; Agilent) with chromatographic signals compared to authentic standards where available. 4MSOB-glutathione, 4MSOB-cysteine and 4MSOB-*N*-acetylcysteine were obtained from Santa Cruz Biotechnology (Dallas, TX, United States). 4MSOB-amine was obtained from Enamine (Monmouth Junction, NJ, United States). 4MSOB-cysteinylglycine was synthesized as described in Schramm et al. (2012). The cyclic 4MSOB-cysteine derivative (2-(4-(methylsulfinyl)butylamino)-4,5-dihydrothiazole-4-carboxylic acid, (Falk et al., 2014), was synthesized as described in Beran et al. (2018) (as “cyclic-Cys conjugate A”). 2PE-amine was obtained from Acros (Geel, Belgium), and raphanusamic acid was purchased from Sigma-Aldrich (St. Louis, Missouri, United States). MRM parameters for parent ion to product ion fragmentation were set as follows (includes parameters used in Gloss et al., 2014; Beran et al., 2018; Chen et al., 2020), and gave the corresponding retention times: *m*/*z* 178.11 →114 (CE, 13 V; DP, 56 V; RT 2.62 min) for 4MSOB-ITC; *m*/*z* 485.11 →179.1 (CE, 29 V; DP, 81 V; RT 2.04 min) for 4MSOB-glutathione; *m*/*z* 356.07 →136.1 (CE, 15 V; DP, 51 V; RT 1.93 min) for 4MSOB-cysteinylglycine; *m*/*z* 299.06 →136.1 (CE, 15 V; DP, 56 V; RT 1.90 min) for 4MSOB-cysteine; *m*/*z* 341.07 →178.1 (CE, 17 V; DP, 56 V; RT 2.22 min) for 4MSOB-*N*-acetylcysteine; *m*/*z* 265.1 →201 (CE, 25 V; DP, 56 V; RT 1.04 min) for 4MSOB-cyclic Cys; *m*/*z* 136 →72 (CE, 17 V; DP, 56 V; RT 0.45 min) for 4MSOB-amine; *m*/*z* 122 →105 (CE, 15 V; DP, 56 V; RT 1.21 min) for 2PE-amine, and *m*/*z* 164 →117.8 (CE, 17 V; DP, 61 V; RT 2.08 min) for raphanusamic acid.

### 2.4 Transcriptional Response of *D. radicum* and *D. floralis* Larvae to 2-Phenylethyl Isothiocyanate

To identify the genes expressed upon ITC exposure, and potentially involved in detoxification of ITC, total RNA was extracted from *D. radicum* and *D. floralis* larvae at the 2nd instar. The larvae were reared on a semi-artificial diet containing milk powder, yeast, freeze-dried turnip, agar (2:2:2:1) and 90% water. The diet of ITC exposed larvae was spiked with 2PE-ITC (0.35 µl of a 6 mmol/ml solution DMSO per g diet, Sigma-Aldrich, St. Louis, Missouri, United States, purity 99%). The larvae, 15 per treatment group and species, were reared on control or ITC diets for 7 days. Diets were refreshed every other day to ensure that the concentration of the ITCs and amines would not decrease too much (Muller et al., 2015). The experiment was set up in a climate cabinet (Percival Scientific, Perry, Iowa, United States) at constant conditions (see above). After 7 days, the remaining larvae were shock frozen at −80°C for 45 min. Then the larval surface was rinsed with autoclaved distilled water before the whole larva was manually crushed in lysis buffer from the ReliaPrep RNA Tissue Miniprep kit (Promega, Madison, United States). Total RNA was extracted from single larvae following the supplier’s recommended protocol. Qualitative and quantitative RNA analyses were performed using gel electrophoresis (1% agarose), a NanoPhotometer^®^ P330 (Implen, Munich, Germany) and a Qubit 2.0 (Invitrogen, Carlsbad, CA/United States, BR RNA kit). We pooled three samples and used three replicates per species and condition (with or without 2PE-ITC). Poly(A)-enriched strand-specific library preparation and RNA sequencing were performed by the Deep Sequencing group of Biotech TU Dresden, Germany on an Illumina NextSeq next-generation sequencer. In total, approximately 570 Mio read pairs having a length of 75 bp were generated.

The recently published reference genome of *D. radicum* (of iDiv_Dra_1.0, GenBank accession number GCA_021234595.1, (Sontowski et al., 2022) was used as a starting point for the expression analysis in both species. To obtain a reference sequence suitable to analyze *D. floralis* expression, we first used Illumina RNAseq reads (NCBI BioSamples: SAMN25131701 and SAMN25131702) of *D. floralis* transcriptomes to polish the existing *D. radicum* reference genome. Reads were first mapped on the iDiv_Dra_1.0 *D. radicum* genome using the Burrows-Wheeler Alignment tool with the BWA MEM algorithm version 0.7.17 (Li and Durbin 2009). The bam file was then passed to Pilon v1.23 (Walker et al., 2014) with default settings, in order to obtain an edited version of the genome more compatible with *D. floralis* (e.g. SNPs and indels with regard to the *D. radicum* genome have been corrected). The existing annotation of iDiv_Dra_1.0 was transferred to this new sequence using the tool liftoff in version 1.6.1 (Shumate and Salzberg 2021). In a second round, and in order to recover larger missing regions in the *D. radicum* reference, all *D. floralis* RNA-seq reads were mapped on the iDiv_Dra_1.0 reference using STAR v2.7.8a (Dobin et al., 2012). All unmapped reads were pulled together and assembled into contigs using Trinity v2.9.1 (Henschel et al., 2012). Those new contigs were annotated with coding regions and UTRs using TransDecoder (version 5.5.0, https://github.com/TransDecoder/TransDecoder). Annotations were filtered for a proper start and end of protein-coding transcripts by applying the GeMoMaAnnotationFilter (GAF, GeMoMa version 1.7.2, Keilwagen et al., 2016; Keilwagen et al., 2018) according to the workflow applied for the annotation of the genome of *D. radicum* (Sontowski et al., 2022). The resulting gene annotations were appended to the liftoff-derived gene annotation file of *D. floralis* (iDiv_DFl_1.0. gff) and accordingly, sequences of the new contigs containing these annotations were added to the set of pilon-derived genome sequences of *D. floralis* (iDiv_DFl_1.0. fasta).

Gene expression was assessed for both species using the nf-core/rnaseq pipeline in version 3.0 (Ewels et al., 2020), which is a standard nextflow pipeline for RNA-seq analysis, relying on STAR v2.6.1d (Dobin et al., 2012) for the alignment and Salmon 1.4.0 (Patro et al., 2017) for the quantification. Gene expression raw counts were used for differential gene expression analysis.

Differential gene expression analysis was performed using DESeq2 v1.3.0 (Love et al., 2014) and both species were treated separately. First, only genes with sufficient counts (e.g. at least 2 samples with more than 5 normalized counts) were retained. Differential expression analysis was then performed using a simple “∼ treatment” design, with 3 replicates for each condition (control and exposed to ITC). Differentially expressed genes were selected based on Benjamini–Hochberg (BH) false discovery rate (FDR) procedure; with a 5% significance threshold. Total number of differentially expressed genes in response to 2PE-ITC containing diet in *D. radicum* and *D. floralis* larvae were presented with a Venn diagram using jvenn (Bardou et al., 2014).

All genes were functionally annotated using PANNZER2 (Protein ANNotation with Z-scoRE, Törönen et al., 2018). Enrichment of GO terms of differentially expressed genes with regard to all expressed genes was tested using the topGO R package in version 2.44.0 (Aibar et al., 2015). Significantly enriched terms within the “Biological Process” and “Molecular Function” families were selected using a Kolmogorov-Smirnov test and a 5% significance threshold.

### 2.5 Validation of Detoxification Candidate Genes in Response to Isothiocyanates Using a Quantitative Gene Expression Approach

From the gene expression data, we performed a targeted screening of differentially expressed genes between the 2PE-ITC and the control group with an adjusted *p*-value <0.05. In the next step, we focused on genes associated with the mercapturic acid conjugation pathway (GSTs, *γ*-glutamyltransferases, dipeptidases, *N*-acetyltransferases) and general detoxification genes (cytochrome P450s, Danielson et al., 1997). From the mercapturic acid pathway, we selected one candidate gene, coding for enzymes from each enzyme class, that was significantly upregulated as a gene of interest. If more candidates fulfilled these expectations, we selected the most reliable candidate gene based on a high number of mapped RNAseq reads and a high log-fold change. Following this procedure, we selected the *CYP6A1* gene as a gene of interest representing the cytochrome P450s, although it did not achieve the targeted *p*-value. To prove that these genes responded specifically to ITCs, we designed qPCR primers for the selected genes (Supplementary Table S1) using the online version of Prime3 v. 4.1.0 (Untergasser et al., 2012). In addition, two primer sets were designed for the housekeeping genes GAPDH and *EF-1α* (Supplementary Table S1). We verified that these genes were stable in their expression over different ITCs and concentrations. We set up an experiment, in which 2^nd^ instar of *D. radicum* and *D. floralis* were fed with a semi-artificial diet containing 0, 1 or 2 µmol 2PE-ITC (0.02, 0.2 or 0.4 µl of a 6 mmol/ml solution in DMSO per g diet) or 4MSOB-ITC (0.2, 2 or 4 µl of a 0.5 mmol/ml solution in ethanol per g diet). To the control diets, either 0.4 µl DMSO or 4 µl ethanol per g diet was added. After 6, 24 and 48 h, we collected 4-5 larvae from each diet condition and each fly species (in total 2 species x 6 conditions x 3 time points x 4-5 replicates per ITC = 150 samples in total). Due to low replicate numbers of *D. floralis* larvae on 2PE-ITC after 48 h, this time point was excluded from the analysis. Larvae were shortly rinsed with tap water to remove soil and diet particles, shock frozen at -80°C, and stored until further use. Single individuals were manually crushed in 500 µl TRIzol™ Reagent (Thermo Fisher Scientific, Schwerte, Germany) and total RNA was extracted following the supplier’s protocol. RNA quantity and quality were assessed using a NanoPhotometer^®^ P330 (Implen, Munich, Germany) and a gel electrophoreses (1% agarose). DNA was digested using DNAse I (Thermo Scientific, Waltham, MA, United States) according to the supplier’s instructions. The quality and quantity of the DNA-free RNA was assessed as described before. This RNA was translated into cDNA using RevertAid H Minus Reverse Transcriptase (Thermo Fisher Scientific, Waltham, MA, United States) according to the supplier’s instructions. Gene expression of candidate genes was examined using qPCR. The qPCR reactions were performed on a CFX384 Touch Real-Time PCR detection system (Bio-Rad, Feldkirchen, Germany) using 5 µl PerfeCTa SYBR Green Supermix (Quantabio, Beverly, MA, United States), 0.5 µl of 10 µM forward and reverse primer (Supplementary Table S1), 3.5 µl water and 1 µl cDNA. The qPCR program was as follows: initial incubation at 95°C for 5 min, followed by 40 cycles at 95°C for 30 s, 58°C for 30 s and 72°C for 30 s, followed by a 0.2°C increment melt curve from 60° to 95°C to confirm that there was a single amplified product representing high specificity. Individual samples were run in triplicate and a negative control with no DNA template was examined for each primer set on each plate. Housekeeping genes and target genes of the same sample were run on the same plate. To compare the expression levels after larval feeding at different ITC concentrations, we calculated the 2^−ΔΔCt^ values.

### 2.6 Effects of Isothiocyanates and Amine Breakdown Products on *D. radicum* and *D. floralis* Performance

To test the effect of ITCs and their breakdown products on the performance of both fly species, we collected 500 eggs from each *Delia* species (*D. radicum* and *D. floralis*) and used 100 eggs per treatment. Eggs from each condition were placed in a plastic box (10 × 10 × 6 cm) filled with autoclaved and moistened sand (Gerhard Rösl GmbH, Jesewitz, Germany). The boxes were closed with a transparent lid and covered with Parafilm. The experiment was set up in a climate cabinet (Percival Scientific, Perry, Iowa, United States) at constant conditions (see section 2.1). After hatching, larvae of the control groups were fed with a semi-artificial diet (see section 2.4) and the ITC groups with the same diet spiked with 2PE-ITC (0.4 µl of a 6 mmol/ml solution in DMSO per g diet, Sigma-Aldrich, St. Louis, Missouri, United States, purity 99%), 4MSOB-ITC (4 µl of a 0.5 mmol/ml solution in ethanol per g of diet, MCE, Sollentuna, Sweden, 98% purity), 2PE-amine (24 µl of a 0.08 mmol/ml solution in DMSO per g diet, 99% purity) or 4MSOB-amine (3.8 µl of a 0.5 mmol/ml solution in ethanol per g diet, 95% purity). In total, we prepared 10 boxes (2 species x 5 conditions with 100 eggs each) including the following conditions: larvae fed on 1) diet without ITC as a control, 2) diet with 2PE-ITC, 3) diet with 4MSOB-ITC, 4) diet with 2PE-amine, and 5) diet with 4MSOB-amine. The diet was replaced with fresh diet every other day and the sand was moistened when needed. The number of larvae and pupae were recorded, and insects were weighed on the 11th, 18th and 25th day after starting the experiment for *D. radicum* and on the 14th, 21st and 31st day for *D. floralis*. Temporal differences between species are based on differences in developmental times. In addition, the number and sex of the emerged adults were determined.

### 2.7 Statistical Analyses Other Than for RNAseq Data

Statistical analyses of data obtained from the GSL-breakdown, ITC-detoxification, performance experiments and qPCR analyses were run on R version 4.03 (R Core Team 2020). Homogeneity of variance across groups was tested using Levene’s test in the “car” package (Fox and Weisberg 2019). Normal distribution of the residuals was assessed visually and with the Shapiro-Wilk test. Data of the ITC breakdown experiment and qPCR data were normalized by log_2_ transformation before analyses. Measured GSL values were statistically compared using a Student’s *t*-Test. The LC-MS/MS peak areas of ITCs, amines and conjugates retrieved in the ITC breakdown experiments were compared using a one-way ANOVA on each compound separately followed by a Tukey post-hoc test. For qPCR data, differences between groups were determined using a two-way ANOVA within each species and gene using time and ITC concentration as independent variables, in combination. Significant differences among conditions within time were identified using Tukey’s “Honest Significant Difference” method as post-hoc test. In the performance experiment, differences between treatments were compared using a log-rank test with the “survival” package 3.2-7 in R (Therneau and Lumley 2015). To test differences in the female-male distribution when larvae fed on different diets, we used a one-proportion *z*-test. The weight of the larvae and pupae were compared within single species and among conditions using a Mann-Whitney test with a Bonferroni correction of the resulting *p*-value.

## 3 Results

### 3.1 Glucosinolates Were Not Degraded by Gut Extracts of *D. radicum* and *D. floralis* Larvae

**FIGURE 1:**
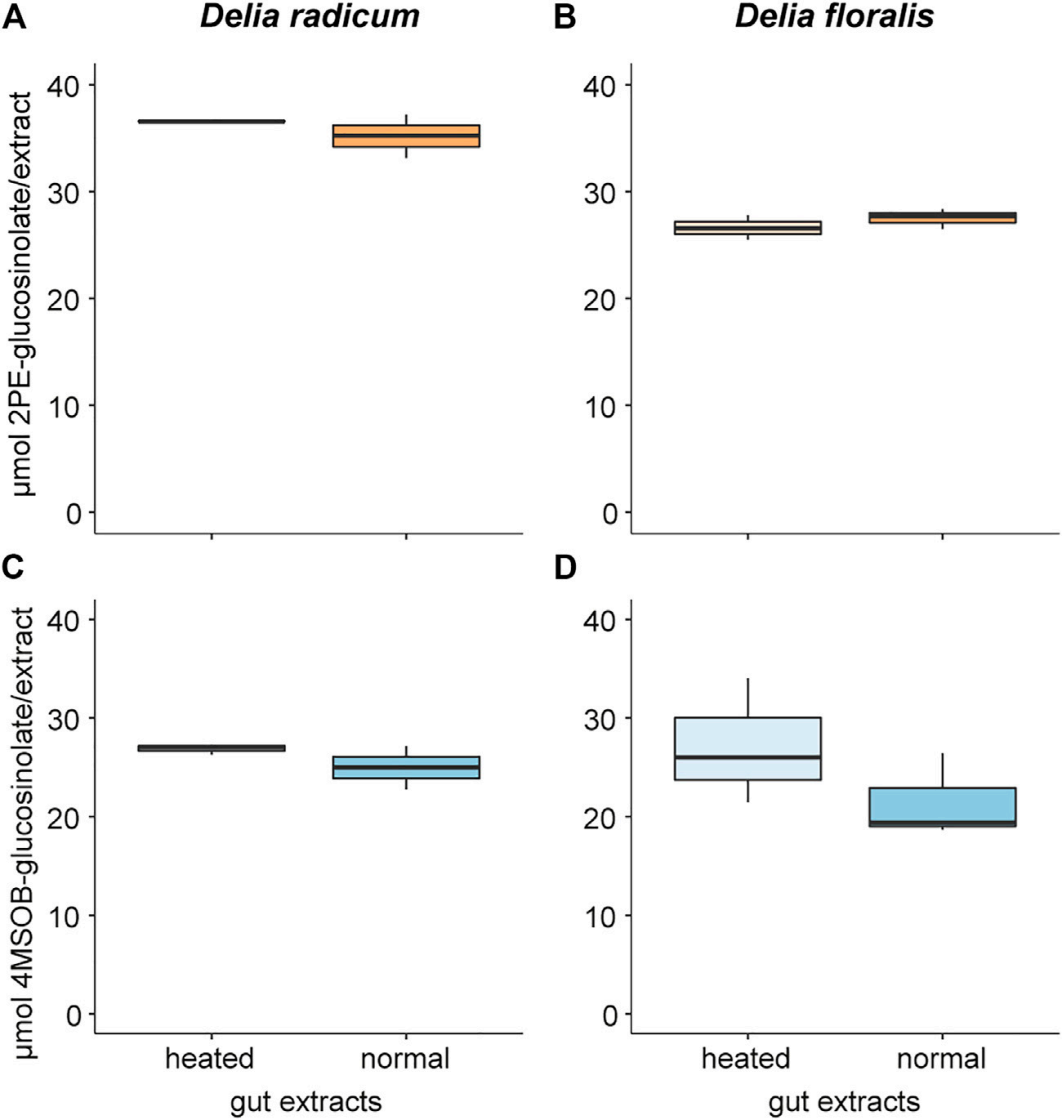
Glucosinolate concentration degraded by gut extracts from *Delia radicum* (left) and *D. floralis* larvae (right). Guts of 2^nd^ instar were extracted and either heated or incubated at room temperature (intact) before adding 2-phenylethyl glucosinolate (2PE-GSL) or 4-(methylsulfinyl)butyl glucosinolate (4MSOB-GSL). **(A)** 2PE-GSL amount in assays containing extracted guts from *D. radicum* and **(B)** from *D. floralis* larvae. **(C)** 4MSOB-GSL amount in assays containing extracted guts from *D. radicum* and **(D)** from *D. floralis* larvae. Each condition was replicated three times with a pool of 10 larval guts.

Neither 2PE-GSL nor 4MSOB-GSL levels differed significantly between incubations with normal and heated larval gut extracts of *D. radicum* and *D. floralis* (Figure 1; Table 1; Supplementary Table S3). We did not detect GSLs in the negative controls containing only extracted guts from *D. radicum* or *D. floralis*, confirming that no residual GSLs were stored in the gut from the diet (Supplementary Table S3).

**TABLE 1:** Comparison of the glucosinolate content in gut extracts of *Delia radicum* or *D. floralis* larvae at room temperature or heated for 7 min at 95°C and thereafter incubated with 4-(methylsulfinyl)butyl or 2-phenylethyl glucosinolates. Each condition was represented by three replicates consisting a pool of 10 larval guts each. *p*-values after *t*-test comparing GSL content in heated versus normal gut extracts.

Species	Glucosinolate	t	Df	*p*-value
*D. radicum*	4-(methylsulfinyl)butyl	1.454	4	0.220
*D. radicum*	2-phenylethyl	1.172	4	0.306
*D. floralis*	4-(methylsulfinyl)butyl	1.279	4	0.270
*D. floralis*	2-phenylethyl	-1.052	4	0.352

### 3.2 Isothiocyanate Was Degraded by Gut Extracts From *D. radicum* and *D. floralis* Larvae

**FIGURE 2:**
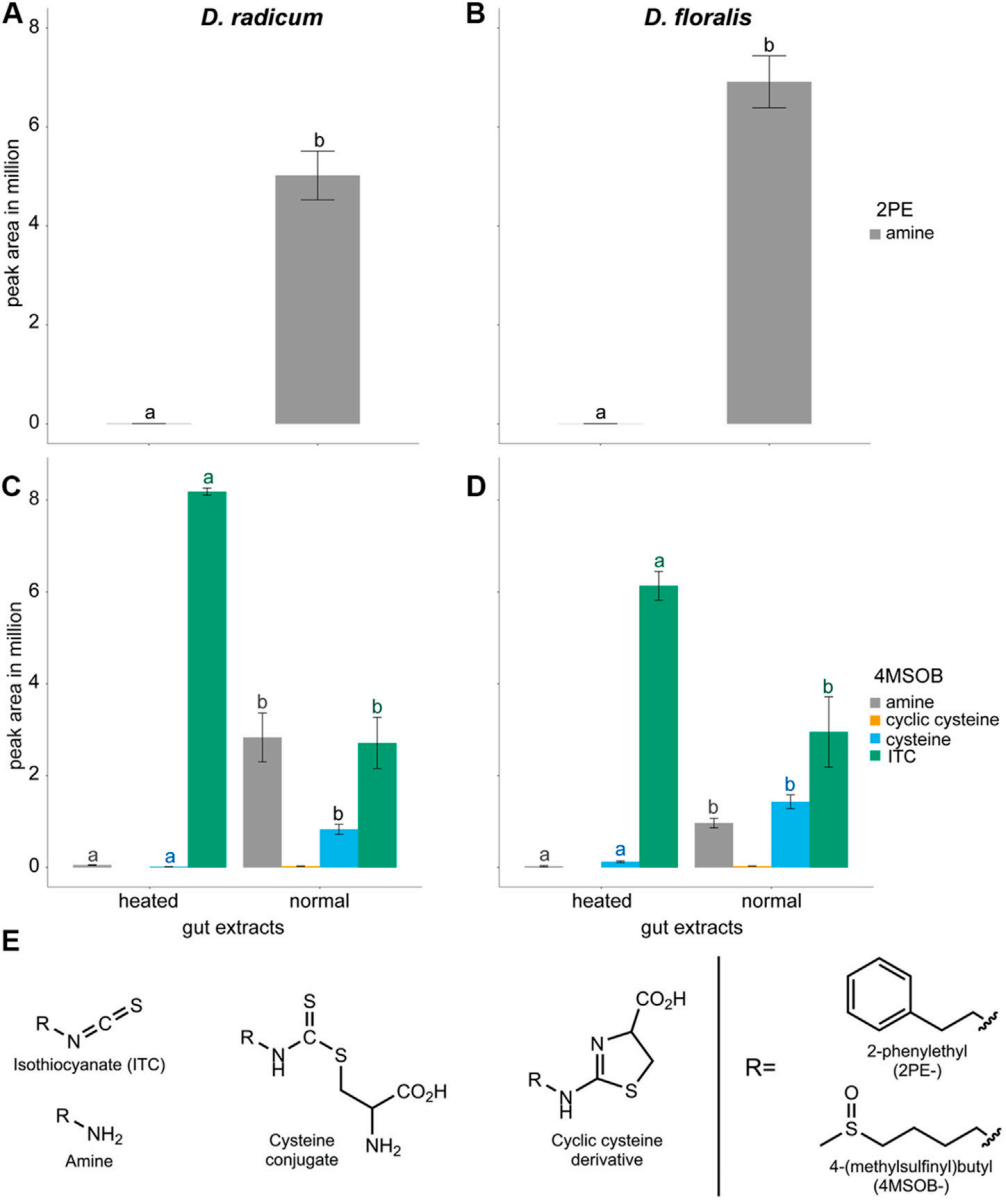
Isothiocyanates (ITCs) and products formed after metabolism by gut extracts from *Delia radicum* (left) and *D. floralis* (right) larvae. **(A)** Blank corrected peak areas of 2-phenylethylamine (PE-amine) formed as a product of 2PE-ITC added to gut extracts of *D. radicum* and **(B)** *D. floralis* larvae. **(C)** Blank corrected peak areas of 4-(methylsulfinyl)butyl-ITC (4MSOB-ITC) and products formed after 4MSOB-ITC was added to gut extracts from *D. radicum* and **(D)** *D. floralis* larvae. **(E)** Structures of compounds detected in *D. radicum* and *D. floralis* gut extracts exposed to ITCs. Guts were extracted and incubated at room temperature (normal) or heated before adding 50 µMol 4MSOB-ITC or 2PE-ITC and incubated for 1 h at room temperature. Each condition was represented by three biological replicates consisting of 10 larval guts. The blank corrected peak areas of each compound were compared between conditions using one-way ANOVA and Tukey post-hoc. Different letters indicate significant differences for each compound (same color for same compound) between the groups within one species (*p* < 0.05).

After incubation with 2PE-ITC, we detected a high proportion of 2PE-amine in the normal gut extracts of both *D. radicum* and *D. floralis* larvae (Figures 2A,B; Table 2; Supplementary Tables S3, S4). Very low levels of this breakdown product were detected in the heated gut extracts of both species. No 2PE-amines were detected in gut extracts without 2PE-ITC (Supplementary Table S3). 2PE-ITC itself does not produce a signal under LC-MS conditions utilized, and 2PE-ITC breakdown products of the mercapturic acid conjugation pathway (PE-glutathione, PE-cysteinylglycine, PE-cysteine, PE-*N*-acetylcysteine) could not be identified due to the lack of authentic standards for these compounds.

**TABLE 2:** One-way ANOVA followed by Tukey post-hoc of the contents of isothiocyanate (ITC) and derived products in normal and heated gut extracts from *Delia radicum* or *D. floralis* larvae incubated with 4-(methylsulfinyl)butyl (4MSOB) or 2-phenylethyl (2PE) isothiocyanate. Each condition was presented by three replicates consisting of a pool of 10 larval guts.

Species	Chemical Product	Heated-Normal Gut Extracts
*D. radicum*	4MSOB-amine	*p* < 0.001 ***
	4MSOB-cysteine	*p* < 0.001 ***
	4MSOB-cyclicCysteine	*p* < 0.001 ***
	4MSOB-ITC	*p* = 0.004 **
	2PE-amine	*p* < 0.001 ***
*D. floralis*	4MSOB-amine	*p* = 0.008 **
	4MSOB-cysteine	*p* < 0.001 ***
	4MSOB-cyclicCysteine	*p* < 0.001***
	4MSOB-ITC	*p* = 0.028 *
	2PE-amine	*p* < 0.001 ***

When incubated with 4MSOB-ITC, normal gut extracts of both species showed reduced levels of this substrate, whereas the ITC breakdown products 4MSOB-amine, 4MSOB-cysteine and cyclic 4MSOB-cysteine (2-(4-(methylsulfinyl)butylamino)-4,5-dihydrothiazole-4-carboxylic acid) were observed in these samples (Figure 2C, D; Table 2; Supplementary Tables S3, S4). In the heated gut samples of both species, 4MSOB-ITC stayed high and only very low levels of 4MSOB-amine and 4MSOB-cysteine were detected, probably as a result of non-enzymatic reactions. Other 4MSOB-conjugates commonly found as products of the mercapturic acid conjugation pathway (4MSOB-glutathione, 4MSOB-cysteinylglycine, 4MSOB-*N*-acetylcysteine), as well as the potential breakdown product of ITC-cysteine conjugates raphanusamic acid, were not detected. Combined these results indicate that *Delia* larval guts possess the enzymatic machinery necessary to break down dietary ITCs.

### 3.3 Gene Expression After Isothiocyanate Exposure Differed Among Isothiocyanates and *Delia* Species

Using both sequence polishing and *de novo* assembly, the reference genome for *D. radicum* was edited to be used as a reference for *D. floralis*. Pilon detected and corrected 81,723 SNPs and indels after aligning 28.5 M of *D. floralis* Illumina RNAseq reads on the *D. radicum* reference genome. *De novo* assembly of the unmapped reads allowed us to reconstruct 36,573 transcripts clustered in 25,064 clusters. In total, 1,948 of those contigs were annotated to contain 2160 protein-coding open reading frames and were added to the edited genome of *D. radicum* to yield a comprehensive genome for *D. floralis* (https://doi.org/10.5281/zenodo.6044094).

RNAseq sequencing yielded on average 46.8 M read pairs for *D. floralis* (min: 41.0 M, median: 47.5 M, max: 52.0 M) and 48.2 M read pairs for *D. radicum* (min: 40.9M, median: 49.8M, max: 53.0 M) per sample. On average, 87.63% of the reads were correctly aligned for *D. floralis* (min: 86.23%, median: 87.68%, max: 88.93%) and 94.44% for *D. radicum* (min: 93.91%, median: 94.52%, max: 94.64%). After filtering of genes with low expression levels, 18,646 genes were considered for differential expression in *D. floralis*, and 26,351 for *D. radicum*.

**FIGURE 3:**
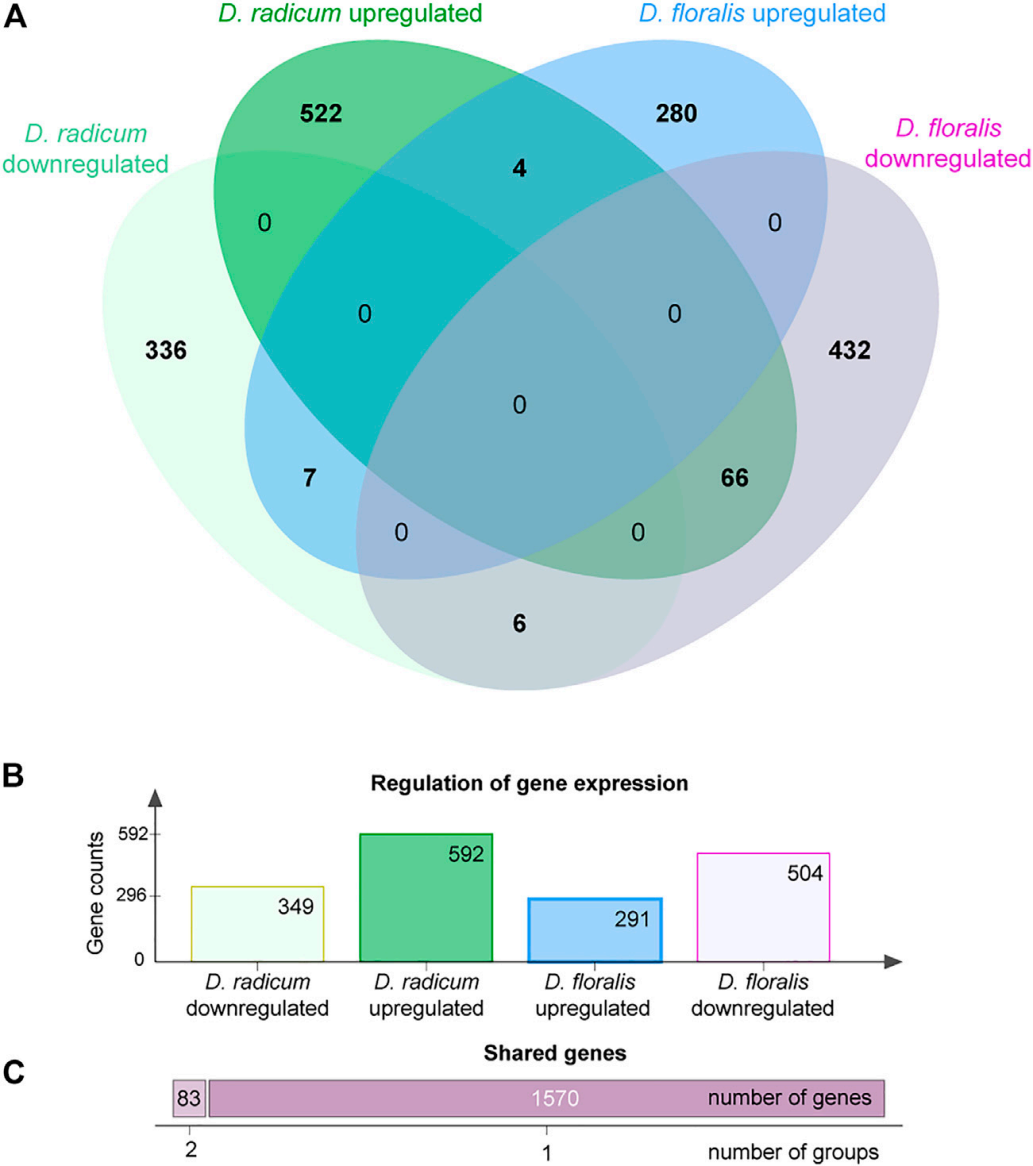
Differentially expressed gene counts in *Delia radicum* and *D. floralis* larvae in response to 2-phenylethyl isothiocyanate (2PE-ITC) in their diet. Larvae were reared on a semi-artificial diet with zero or 2 µmol 2PE-ITC/g diet for 7 days. Genes were counted as up- or downregulated with a *p*-value < 0.05 after FDR. **(A)** Venn-diagram representing the differentially expressed gene counts between both species. **(B)** Barplot of gene counts of up- and downregulated genes per species. **(C)** Number of genes responding to 2PE-ITC in one group (dark purple, right bar) or differentially expressed shared by two species-response-direction combinations (light purple, left bar).

**FIGURE 4:**
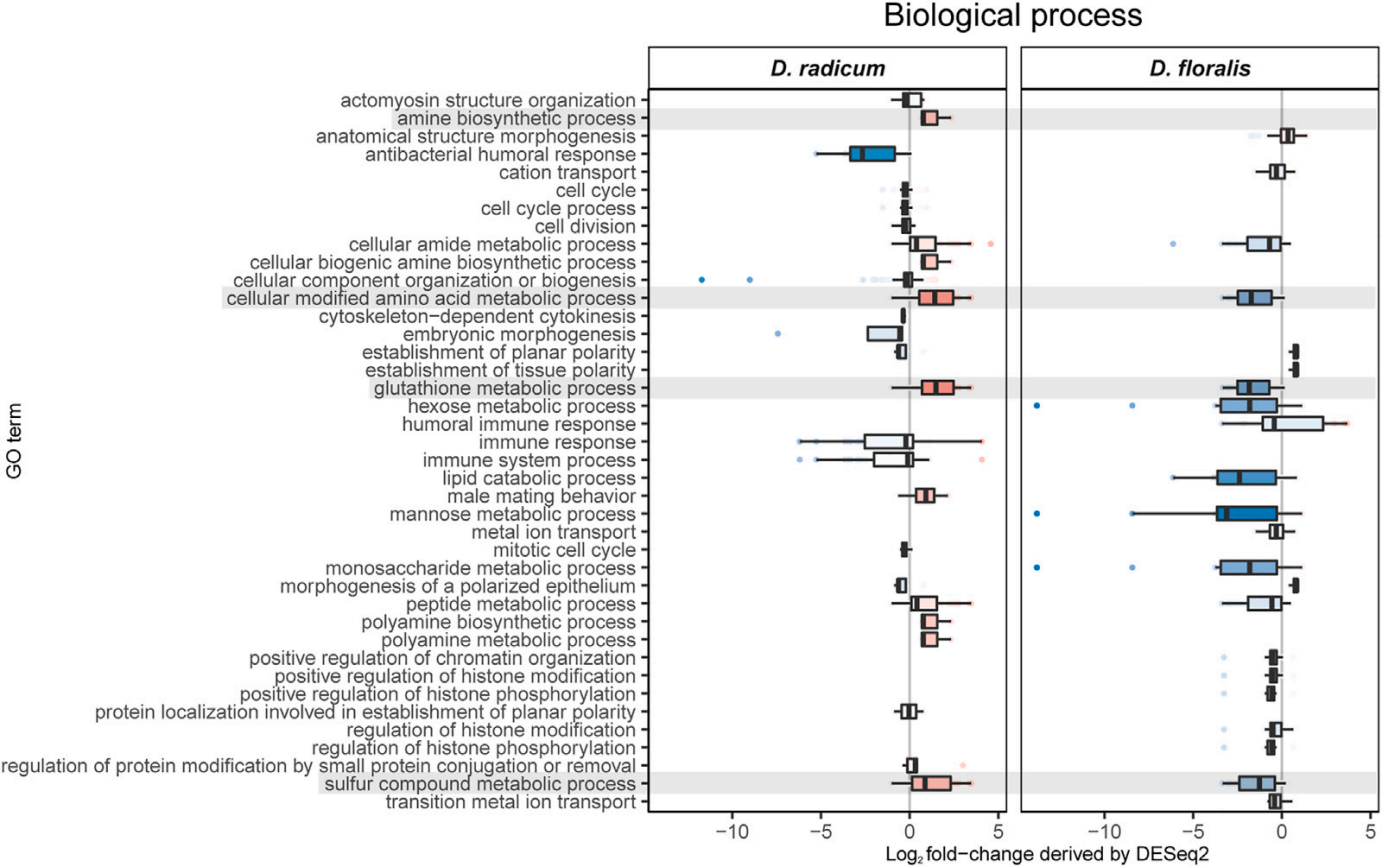
Gene ontology analyses of biological processes based on differently expressed GO terms in *Delia radicum* and *D. floralis* larvae exposed to 2-phenylethyl isothiocyanate (2PE- ITC) in their diet. Box plots show the distribution of Log_2_ fold-changes. Results are presented by medians (horizontal bar), interquartile ranges (IQRs; boxes), and data ranges (whiskers) excluding outliers (defined as > 1.5 x IQR). Rows (boxes) are labeled by GO terms. Only GO terms that were significantly differentially observed between control and 2PE-ITC in a GO-enrichment analysis (*p* < 0.05) are considered. Red colors: upregulated, blue colors: downregulated, Intensity of color: strength of up- or downregulation, grey color: GO terms of interest.

We found 592 upregulated and 349 downregulated genes in *D. radicum* larvae when exposed to 2PE-ITC (Figure 3). The reverse was found in *D. floralis* larvae, where more genes were downregulated (504) than upregulated (291). Only four genes were up- and six downregulated in both species. More genes (in total 73) were upregulated in one species and downregulated in the other. Overall, genes of the same gene ontology (GO) term classes responded to ITCs in the larvae of both species, but in different directions (Figure 4, Supplementary Figure S1). While upregulated genes in *D. radicum* corresponding to 22 GO term classes, only six corresponding classes were observed to respond in *D. floralis*. Upregulated GO-classes and genes in *D. radicum* were mainly involved in glutathione, amino acid, peptide, sulfur compound and amine metabolic processes as well as sulfur compound binding (Figure 4; Supplementary Figure S1). The gene expression in *D. floralis* larvae comprised metamorphosis-related terms and whereas the glutathione, amino acid and sulfur compound metabolic processes were downregulated (Figure 4). Zooming in to the genes connected to the mercapturic acid conjugation pathway in *D. radicum* larvae revealed eight significantly upregulated genes encoding for GSTs, one for *γ*-glutamyltransferases, two for dipeptidases and five for *N*-acetyltransferases (Supplementary Table S1). We identified one significantly downregulated gene encoding for a GST and one for an *N*-acetyltransferase. In *D. floralis* larvae we found no significantly upregulated genes from any of these enzyme classes; actually, we found three downregulated GSTs and four *N*-acetyltransferases in this species.

**FIGURE 5:**
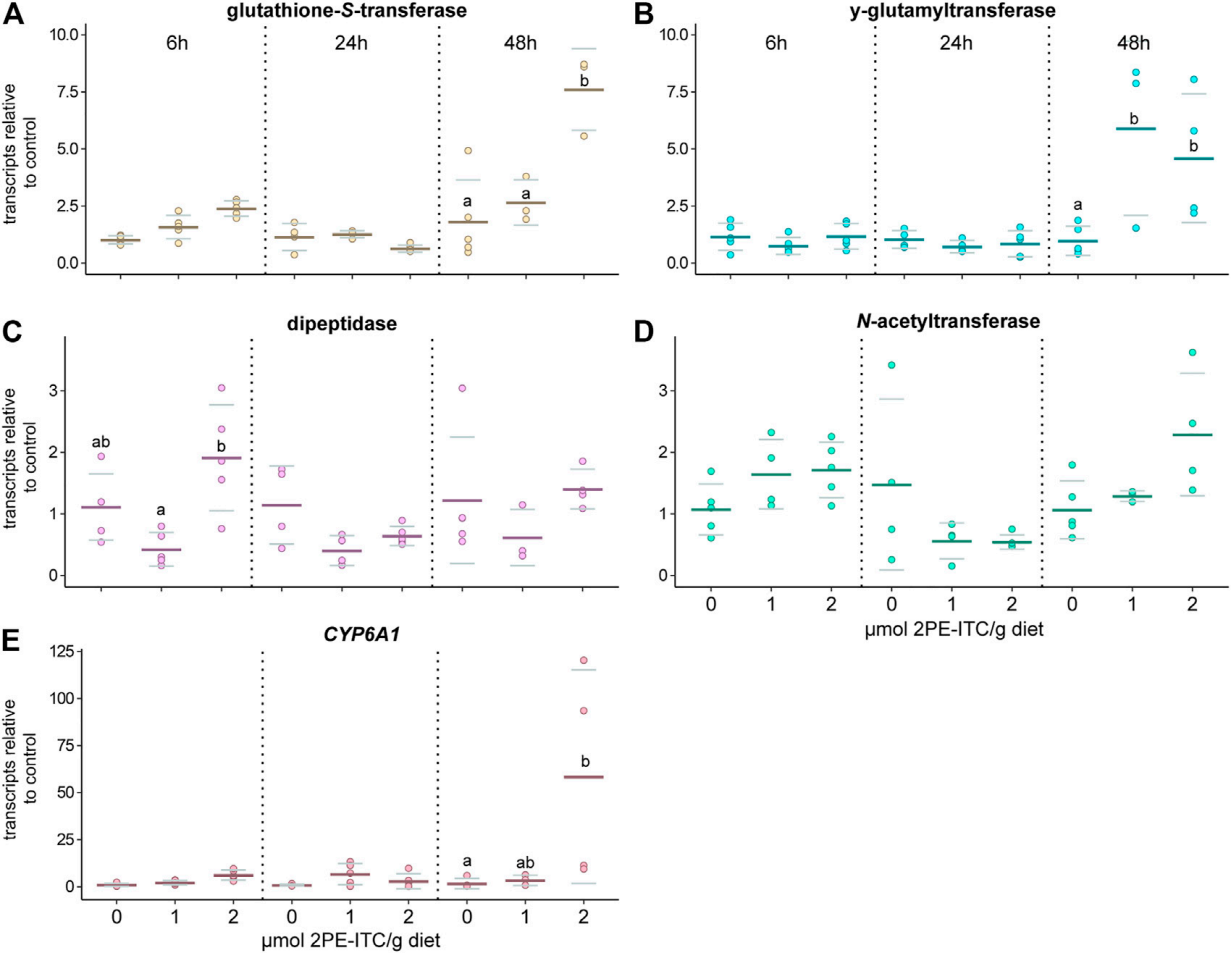
Expression of selected candidate genes related to the mercapturic acid pathway **(A–D)** and cytochrome P450 (CYP6A1, E) responding to 2-phenylethyl isothiocyanate (2PE-ITC) in *D. radicum* larvae measured by qPCR. Larvae were fed on 0, 1 or 2 µmol 2PE-ITC/g diet for 6, 24 and 48 h and the response of transcripts of a candidate gene coding for glutathione-*S*-transferase **(A)**, *γ*-glutamyltransferase **(B)**, dipeptidase **(C)**, *N*-acetyltransferase **(D)** and CYP6A1 **(E)** compared relative to the control sample using 2^−ΔΔCt^. Mean values were presented as dark lines, standard deviation as grey lines and individual values as dots. Different letters indicate significant differences between the treatments of one timepoint with a *p*-value < 0.05 using two-way ANOVA followed by Tukey post-hoc test.

The qPCR analyses showed that the selected candidate genes of the GST, *γ*-glutamyltransferase and cytochrome P450 (CYP6A1) family were upregulated in *D. radicum* larvae that were exposed to 2 µmol 2PE-ITC for 48 h (Figure 5A,B,E; Table 3; Supplementary Table S4). The expression of the selected genes for dipeptidase and *N*-acetyltransferase did not change upon ITC exposure (Figure 5C,D; Table 3; Supplementary Table S4). We found no differential expression of any selected detoxification genes in *D. floralis* feeding on 2PE-ITC containing diets (Supplementary Figure S2; Table 3; Supplementary Table S4). None of the selected candidate genes responded to 4MSOB-ITC in either species at any concentration or time point, (Supplementary Figures S3,S4, Table 3; Supplementary Table S4).

**TABLE 3:** Two-way ANOVA and Tukey post-hoc of transcripts in *Delia radicum* or *D. floralis* larvae treated with different ITCs at different time points (6, 24, 48 h), relative to untreated controls. Each condition and time point were represented by 4 till 5 replicates, depending on the survival of the larvae. 2PE-ITC (2-phenylethyl isothiocyanate), 4MSOB-ITC (4-(methylsulfinyl)butyl isothiocyanate).

Gene	glutathione-S*-transferase*	*γ-glutamyl-transferase*	*dipeptidase*	N*-acetyl-transferase*	CYP6A1
*D. radicum*					
2PE-ITC					
Time	*p* < 0.001 ***	*p* < 0.001 ***	*p* = 0.117	*p* < 0.001 ***	*p* = 0.108
Condition	*p* < 0.001 ***	*p* = 0.218	*p* < 0.001 ***	*p* = 0.443	*p* < 0.001 ***
Time: Condition	*p* < 0.001 ***	*p* = 0.003 **	*p* = 0.238	*p* = 0.052	*p* = 0.022 *
4MSOB-ITC					
Time	*p* = 0.034 *	*p* = 0.881	*p* = 0.064	*p* = 0.070	*p* = 0.046 *
Condition	*p* = 0.207	*p* = 0.030 *	*p* = 0.225	*p* = 0.284	*p* = 0.061
Time: Condition	*p* = 0.056	*p* = 0.794	*p* = 0.453	*p* = 0.287	*p* = 0.538
*D. floralis*					
2PE-ITC					
Time	*p* = 0.546	*p* = 0.708	*p* = 0.358	*p* = 0.203	*p* < 0.001 ***
Condition	*p* = 0.509	*p* = 0.528	*p* = 0.014 *	*p* = 0.155	*p* < 0.001 ***
Time: Condition	*p* = 0.829	*p* = 0.408	*p* = 0.889	*p* = 0.791	*p* < 0.001 ***
4MSOB-ITC					
Time	*p* = 0.015 *	*p* = 0.001 **	*p* < 0.001 ***	*p* = 0.002 **	*p* < 0.001 ***
Treatment	*p* = 0.007 **	*p* = 0.714	*p* = 0.076	*p* = 0.247	*p* < 0.001 ***
Time: Condition	*p* = 0.382	*p* = 0.146	*p* = 0.228	*p* = 0.038 *	*p* < 0.001 ***

### 3.4 Isothiocyanates in the Diet Reduce *D. radicum* and *D. floralis* Performance More Than Their Breakdown Products

**FIGURE 6:**
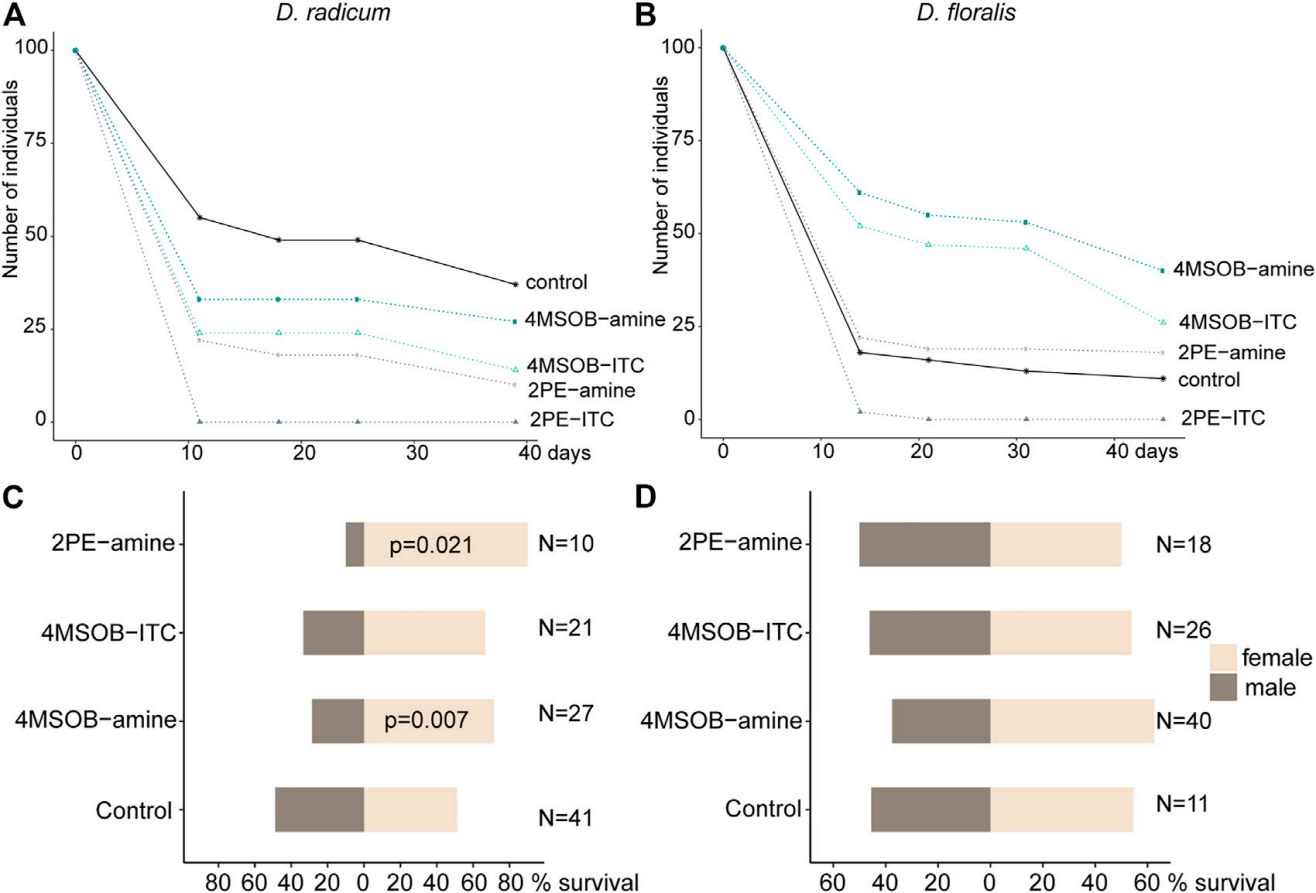
Effects of isothiocyanates (ITCs) and amines on *Delia radicum* and *D. floralis* performance. Larvae were fed on a semi-artificial diet (control) or the same diet spiked with 2 μmol/g diet of 2-phenylethyl isothiocyanate (2PE-ITC), 2PE-amine, 4-(methylsulfinyl)butyl isothiocyanate (4MSOB-ITC), or 4MSOB-amine per g diet. **(A)** Survival of *D. radicum* and **(B)** *D. floralis* over 40 days are presented as Kaplan-Meier curves. **(C)** Male-female ratios in the resulting *D. radicum* and **(D)** *D. floralis* adults compared using a one-proportion *z*-test. Significant *p*-values below 0.05 are included in figure. *N* = number of males and females.

In order to determine the possible advantages of metabolizing 2PE- and 4MSOB-ITCs to *Delia* larvae, we fed ITCs and their major amine detoxification products to the insects in a semi-artificial diet. In general, consuming 4MSOB-ITC, 2PE-ITC, 4MSOB-amine or 2PE-amine all negatively affected the survival rate of *D. radicum* (Figure 6A; Table 4). Among these conditions, larvae performed markedly better when feeding on a diet containing the detoxification product 2PE-amine than on those with the toxin 2PE-ITC. A similar trend (*p* > 0.05) was detected in the 4MSOB-ITC and 4MSOB-amine treatments. Additionally, *D. radicum* larvae survived better when feeding on 4MSOB-ITC than on 2PE-ITC-containing diet; in fact, none of the larvae reached the second instar when fed on 2PE-ITC containing diets.

**TABLE 4:** Log-rank-test of the survival in *D. radicum* and *D. floralis* feeding on a semiartificial diet with 2-phenylethyl isothiocyanate (2PE-ITC), 2-phenylethylamine (2PE-amine), 4-(methylsulfinyl)butyl isothiocyanate (4MSOB-ITC), 4-(methylsulfinyl)butylamine (4MSOB-amine) or without ITCs or amines (control) using.

	*D. radicum*		*D. floralis*	
Condition	*p*-value	χ^2^	*p*-value	χ^2^
Control–2PE-amine	<0.001 ***	48.4	0.4	0.6
Control–2PE-ITC	<0.001 ***	115	<0.001 ***	15.2
2PE-amine–2PE-ITC	<0.001 ***	28.2	<0.001 ***	20.6
Control–4MSOB-amine	<0.001 ***	35.3	<0.001 ***	65.9
Control–4MSOB-ITC	<0.001 ***	44.4	<0.001 ***	38.5
4MSOB-amine–4MSOB-ITC	0.3	0.9	0.002 **	9.6
2PE-amine–4MSOB-amine	0.2	1.7	<0.001 ***	56.9
2PE-ITC–4MSOB-ITC	<0.001 ***	31.4	<0.001 ***	82.4

While larval weight gain was not affected by exposure to the different compounds, *D. radicum* pupae were slightly heavier when the larvae had been raised on diet containing 4MSOB-amine (Supplementary Figure S5). Additionally, the consumption of 4MSOB-amine and 2PE-amine affected the sex ratio of the emerging adults; diets with amines shifted the female-male ratio towards an increased proportion of females (Figure 6C; Supplementary Table S5).

*D. floralis* performed similarly to *D. radicum* when exposed to 2PE-ITC; none of the *D. floralis* larvae reached the 3rd instar when fed 2PE-ITC containing diet. We also found a higher survival of larvae fed on diets with amine compare to those fed on ITC (Figure 6B; Table 4). Surprisingly, the presence of 4MSOB-ITC or 4MSOB-amine resulted in a more than two-fold larger survival rate for *D. floralis* larvae relative to the larvae fed on control diet.

Although several of the growth effects of ITCs and amines were similar between both fly species, we observed species-specific effects on larval and pupal weights. While the larval weight of *D. floralis* decreased during the consumption of 4MSOB-amines, the pupal weight increased when consuming 4MSOB-ITCs (Supplementary Figure S5). Species-specific effects were also detected in the female-male ratio: contrary to *D. radicum*, no effect of these chemicals was detected on the sex ratio of emerging *D. floralis* (Figure 6D; Supplementary Table S5).

## 4 Discussion

**FIGURE 7:**
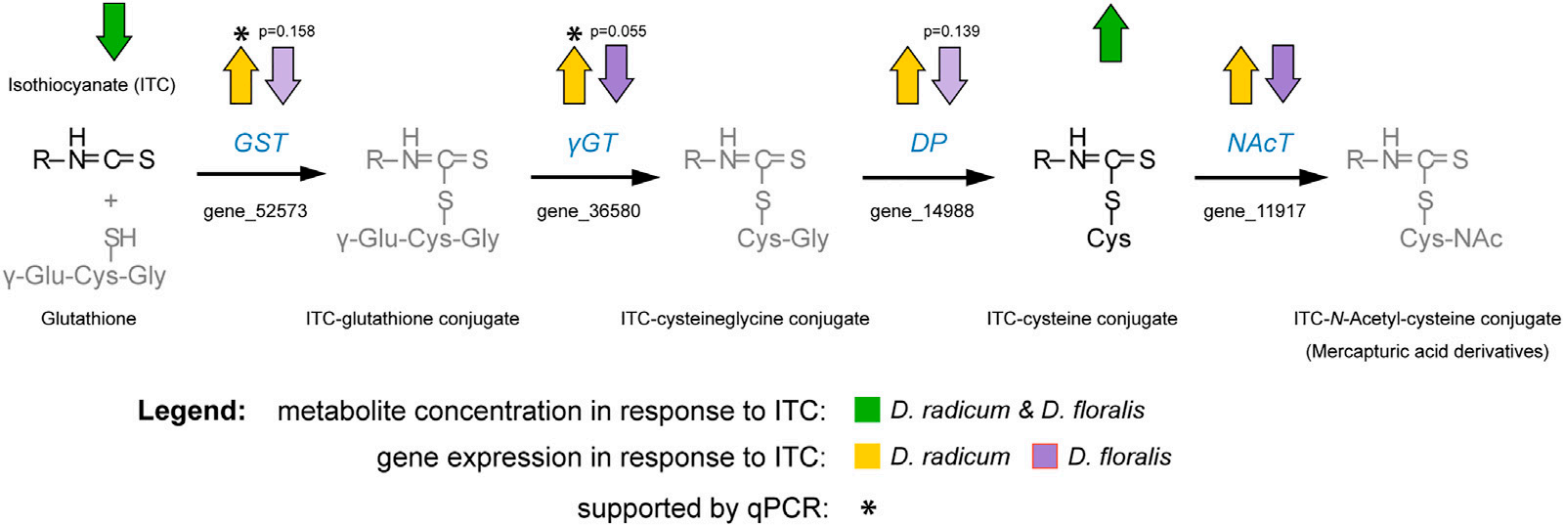
Schematic illustration of the observed detoxification of isothiocyanates (ITCs) in the larvae of *Delia radicum* and *D. floralis* combined with the regulation of genes in the mercapturic acid conjugation pathway as assessed with targeted metabolomic and transcriptomic data. Arrows indicate an up- or downregulation with a *p*-value < 0.05 unless otherwise noted in the figure. Pathway was modified after Higdon et al. (2007). Abbreviations: GST, glutathione-*S*-transferase; γGT, gamma-glutamyltransferase; DP, dipeptidase; NAcT, *N*-acetyltransferase.

Herbivores have to overcome several challenges to thrive on chemically-defended plant tissues. Here, we examined how two herbivores specialized on *Brassica* roots, the larvae of *D. radicum* and *D. floralis*, have adapted to the chemical defense system of their host plants. We found that larvae from both species possess the enzymatic machinery necessary to neutralize the toxic hydrolysis product (ITCs), rather than transforming GSLs which are their precursors. Previous studies have reported that *Brassica* plants release ITCs upon *D. radicum* feeding (Crespo et al., 2012) which corroborates our observation that these insects cannot prevent ITCs from being formed. Despite the fact that *D. floralis* and *D. radicum* are closely related, share a similar host plant range and feeding mode, and elicit similar responses in their host plants (Sontowski et al., 2019), they seem to use different mechanisms to overcome the ITCs. Our experiments showed that gut extracts from both species produced 2PE-amine when incubated with 2PE-ITC. After adding 4MSOB-ITCs to gut extracts, we detected three breakdown products, the 4MSOB-ITC-cysteine conjugate, a cyclic 4MSOB-ITC-cysteine product formed by intramolecular cyclization of the linear 4MSOB-ITC-cysteine conjugate (Falk et al., 2014), and 4MSOB-amine. The latter amine appears to be formed directly via hydrolysis of the corresponding ITC, and not through metabolism of 4MSOB-cysteine, as raphanusamic acid could not be detected as a co-occurring product. The formation of cysteine conjugates indicated that the mercapturic acid pathway is activated for ITC detoxification in both insect species (Figure 7). Indeed, the expression of two potential mercapturic acid pathway related genes was upregulated in *D. radicum* feeding on 2PE-ITC enriched diet (Figure 7). In addition, we identified one candidate, the cytochrome CYP6A1, representing a more general detoxification gene family, which was also upregulated in *D. radicum* larvae exposed to 2PE-ITC, but not in those fed with 4MSOB-ITC. Neither of these genes was upregulated in *D. floralis*. This led to the conclusion that the regulation of detoxification genes in these closely related congeneric root herbivores is controlled in an ITC-specific and potentially in a species-specific fashion. Lastly, we tested whether the amines are true detoxification products, i.e. have fewer negative effects on *Delia* performance relative to their parent compounds. Larvae performed significantly better when consuming amines than ITCs for both 4MSOB- and 2PE-ITC. This means that the amines indeed are less toxic than ITCs and thus can be considered detoxification products. Interestingly, amine exposure caused a species-specific shift in sex ratios of *D. radicum* to more females whereas no such effect was detected in *D. floralis*.

In general, there are three points in the GSL-myrosinase system at which insects can interfere to reduce its defensive effect: structural changes to the GSL precursor, shifting the myrosinase reaction towards less toxic products, or dealing with the ITCs after they have been formed (Wittstock et al., 2003). Our study shows that belowground feeding *Delia* larvae follow the third strategy. Enzyme preparations from both fly species *D. radicum* and *D. floralis* metabolize ITCs via the mercapturic acid conjugation pathway to form ITC-cysteine conjugates. This detoxification mechanism is commonly found in a wide range of insect herbivores and mammals (Wadleigh and Yu 1988; Schramm et al., 2012; Beran et al., 2018), including flies where it was found in several *Drosophila* species (Gloss et al., 2014). In addition, we found a second breakdown product, 4MSOB-/2PE-amine. ITC-derived amines and/or the enzymes responsible for their formation have also been detected in the flea beetle *P. chrysocephala* (Beran et al., 2018). In addition, they can be produced by microbes, such as the phytopathogenic fungus *Sclerotinia sclerotiorum*, pathovars of the bacterium *Pseudomonas syringae*, and microbial isolates from the gut of *D. radicum* (Fan et al., 2011; Welte et al., 2016; Beran et al., 2018; van Den Bosch et al., 2018; Chen et al., 2020). However, it is still unclear whether the ITC-derived amines excreted by insects are the result of ITC hydrolysis by the insect’s enzymes or are produced by their associated (gut) microbiota. Since *Delia* larvae feed on roots, they may take up bacteria or fungi carrying ITC hydrolases from the rhizosphere. These microbes are may be enriched in the rhizosphere of *Brassica* species, because ITC and GSL breakdown products are excreted in root exudates (van Dam and Bouwmeester 2016). The presence of ITCs in the rhizosphere may select for microbes with ITC detoxification enzymes. Whether the larvae benefit from the presence of such microbes and may “split the costs” associated with detoxifying ITCs remains an open question.

Based on the production of 4MSOB-ITC derivatives, we found that both fly species (including their associated microbiomes) produced similar ITC breakdown products but tend to be in different proportions. Based on HPLC-MS/MS peak areas alone, gut extracts of *D. radicum* produced larger signals for 4MSOB-amine than those detected for 4MSOB-cysteine conjugates, whereas the corresponding signals detected for these conversion products had similar intensities in assays using *D. floralis* gut extracts. Species-specific results were observed in 2PE-ITC metabolism in gut extracts. In these samples, the formation of 2PE-amine was higher in larval gut extracts from *D. floralis* compared to *D. radicum*. This indicates that both species use the same pathways (mercapturic acid and hydrolytic), but might prioritize these differently, possibly caused partly by different gut microbial communities in both species. Unfortunately, due to the lack of ionization of the 2PE-ITC under LC-MS conditions and the lack of appropriate authentic chromatographic/mass spectrometric standards for its conjugates, we could not investigate the formation of mercapturic acid products derived from 2PE-ITC via HPLC-MS/MS. Therefore, further studies are necessary to conclude whether the formation of breakdown products is ITC-specific and whether the proportion of metabolism through the direct hydrolyzation or conjugation differs.

Using comparative RNAseq analysis, we identified several gene families and gene candidates coding for detoxification-related enzymes that are regulated upon ITC exposure. Based on our metabolite results, we directed particular interest to gene families encoding enzyme classes associated with the mercapturic acid conjugation pathway. GSTs are the starting point of the mercapturic acid pathway, conjugating ITCs to glutathione. This step is followed by stepwise hydrolysis of the amino acids of which glutathione is composed (Habig et al., 1974). These amino acids are potentially reabsorbed by the herbivore. This conjugation increases the water solubility of the ITCs and other xenobiotics and facilitates their excretion (Field and Thurman 1996). This pathway has been described in several Brassicaceae-feeding herbivores which deploy this pathway to degrade ITCs (Schramm et al., 2012; Gloss et al., 2014; Beran et al., 2018). In more detail, GSTs conjugate ITCs to glutathione, followed by a conversion into ITC-CysGly by *γ*-glutamyltransferases. In the next step, ITC-cysteine conjugates are formed thanks to the activity of dipeptidases. These ITC-cysteine conjugates are further *N*-acetylated in some species (Figure 7) (Higdon et al., 2007). We found that cysteine conjugates of 4MSOB-ITC were formed in gut extracts of both fly species after externally adding 4MSOB-ITCs. In parallel, we found an upregulation of genes encoding for GST and *γ*-glutamyltransferases in *D. radicum* larvae, which were fed on 2PE-ITC-containing diet after 48 h. In *D. floralis* the expression of these particular genes was not upregulated by any tested ITCs at the two tested time points (6 and 24 h). The putative detoxification gene *CYP6A1*, belonging to the large gene family of cytochrome P450 monooxygenases (P450s), was strongly upregulated in *D. radicum* feeding on 2PE-ITCs as well. P450s are generally involved in the adaptation of insects to synthetic insecticides and host plant allelochemicals (Rose et al., 1991; Feyereisen 1999; Chiu et al., 2008; Smith et al., 2016). This suggests that *CYP6A1* might be involved in a more general GSL/ITC detoxification process in *D. radicum*. Although the RNAseq data revealed changes in the expression profile of the *CYP6A1* gene in *D. floralis* (Supplementary Table S1), we detected no expression in the qPCR analysis. Despite the qPCR primer sequences of the other selected detoxification genes were expressed in both species, the CYP6A1 primer sequence was not expressed in *D. floralis* (Supplementary Figure S6). Selecting new primer regions may result in qPCR signals. These findings suggest that the expression of potential detoxification gene fragments seems to be species-specifically regulated in *Delia* species.

The RNAseq data also revealed more global differential gene expression patterns in response to ITC exposure. Whereas *D. radicum* upregulated many genes when exposed to ITCs, *D. floralis* downregulated a large number of genes involved in primary metabolic and detoxification processes. The systematic downregulation of genes in *D. floralis* may be caused by a lower tolerance level of these larvae to 2PE-ITC resulting in broader toxicity effects of ITCs that affect metabolism more generally than in *D. radicum*. Whether *D. radicum* larvae prefer plant roots with high 2PE-GSL levels and *D. floralis* larvae roots with a different GSL profile can only be hypothesized. Species- and even population-specific preferences to different host plants within Brassicaceae have been described in herbivores including *D. radicum* and Pieridae (Van Leur et al., 2008; Newton et al., 2010; Lamy et al., 2018; Okamura et al., 2019).

An explanation for the lack of putative gene expression response to 4MSOB-ITC in both species may be that we selected gene candidates based on their expression in response to 2PE-ITC. In case of ITC-specific gene activation, which is suggested by our data, we may have thus missed 4MSOB-ITC specifically expressed genes. A second explanation might be that 4MSOB-ITC is simply less poisonous than 2PE-ITC to the larvae of both *Delia* species. Our performance data indeed showed that the larval performance is less affected by 4MSOB-ITC than by 2PE-ITC, and in case of *D. floralis*, the former may even enhance survival. This may mean that the larvae do not need to express their detoxification machinery to neutralize this compound. While this assumption would contradict our detection of 4MSOB-cysteine in both *Delia* species, it would be supported by the relatively higher survival of both species when feeding on 4MSOB-ITC/amine than on 2PE-ITC/amine. A third explanation is that under natural conditions the larvae are rarely confronted with 4MSOB-ITC. The precursor of 4MSOB-ITC is 4MSOB-GSL. This is the major leaf GSL in *Arabidopsis thaliana* ecotype Columbia-0, as well as in some varieties of cultivated Brassicas such as broccoli and Brussels sprouts, and therefore well studied and commercially available (Gross et al., 2000). In the main host plants of *D. radicum* and *D. floralis*, 4MSOB-GSL is present at high concentrations in seeds, sprouts and leaves, but only in low concentrations in their roots (Guo et al., 2014; Bhandari et al., 2015). The precursor of 2PE-ITC is 2PE-GSL, which is one of the main GSLs in the roots of many *Brassica* species (Van Dam et al., 2009). This may mean that the larvae are more adapted to deal with 2PE-ITC.

On the organismal level it is known that ITCs can impair the survival and development of specialist herbivores (Agrawal and Kurashige 2003; Sun et al., 2019). For instance, allyl-ITC reduced the survival and growth of the cabbage white, *Pieris rapae* (Agrawal and Kurashige 2003). We also found a negative effect of 2PE-ITC on the survival rate of both *D. radicum* and *D. floralis*. In addition, 2PE-ITC can successfully defend roots against nematodes, in particular *Pratylenchus penetrans* (Potter et al., 1998; Kabouw et al., 2010) and wireworms (*Limonius infuscatus*; Brown et al., 1991). Here we found that it can also defend plants against *D. radicum* and *D. floralis* larvae. The second ITC tested, 4MSOB-ITC, had a much weaker negative effect, if at all. Interestingly, 4MSOB-ITC and in particular its amine even increased the performance of *D. floralis*. Compared to ITCs, both species performed better on the corresponding amines. This finding suggests that ITCs were properly detoxified by the ITC hydrolysis reactions, and thus may be an adaptative mechanism by which the larvae of *Delia* spp. overcome their host plant’s defense system.

In conclusion, we could successfully characterize how gut extracts from two belowground *Brassica* specialists, *D. radicum* and *D. floralis*, detoxify their host plant’s defense system. Both species (including their microbiomes) detoxify ITCs to ITC-cysteine conjugates and amines using the mercapturic acid conjugation pathway and a hydrolytic pathway, respectively. In spite of producing similar detoxification products, their close phylogenetic relationship, and overlapping host plant range, the two species do not deploy the same enzymatic mechanisms in the detoxification process. Especially on the level of gene expression and performance, *D. radicum* and *D. floralis* respond differently to ITC exposure. Along with species-specific effects, the two herbivores also responded differently to ITCs with different side-chains: *D. radicum* and *D. floralis* both were most susceptible to 2PE-ITCs. Such differences might also explain different host plant preferences within the Brassicaceae family (Lamy et al., 2018). This knowledge might be considered in the selection of lines to breed more resistant or less attractive crops using natural variation for GSL and ITC production in *Brassica* accessions and species (van Dam et al., 2012; Sontowski et al., 2019). The putative detoxification genes/gene families we identified may also serve as a starting point for further studies aiming to develop RNAi-based pest management strategies.

## Data Availability

The datasets generated and analyzed in this study can be found in the National Center for Biotechnology (NCBI, https://www.ncbi.nlm.nih.gov). BioSample metadata are available in the NCBI BioSample database (http://www.ncbi.nlm.nih.gov/biosample/) under accession numbers SAMN25131701 and SAMN25131702 for *D. floralis* and SAMN19640657 and SAMN19640660 for *D. radicum*. Corresponding raw read files are available in the NCBI sequence read archive (https://www.ncbi.nlm.nih.gov/sra) under accession numbers: SRS11844094, SRS11844093, SRS9199199, and SRS9199198. Analyzed genome sequences of *D. radicum* are available via NCBI under the GenBank accession number GCA_021234595.1. Genome sequences of *D. floralis*, corresponding gene annotation and functional annotation, unmapped-read-assembly results, expression values, and DESeq2 results are available via Zenodo (https://doi.org/10.5281/zenodo.6044094).
