# Supplementary material for: Mechanisms of Isothiocyanate Detoxification in Larvae of Two Belowground Herbivores, Delia radicum and D. floralis (Diptera: Anthomyiidae)
Source: Front Physiol. 2022 Apr 29;13:874527. doi: 10.3389/fphys.2022.874527 (PMC9098826; doi:10.3389/fphys.2022.874527)
Supplement: Supplementary file 2 [file DataSheet1.docx]

Supplementary Material

Supplementary Table 1: Selected candidate genes of the mercapturic acid conjugation pathway

Supplementary Table 2: Primers

Supplementary Table 3: Glucosinolates, isothiocyanates and breakdown products in gut extracts

Supplementary Table 4: ANOVA of the isothiocyanate breakdown and qPCR experiment

Supplementary Table 5: Female-male distribution of the performance experiment

Supplementary Figure 1: GO terms of molecular functions

Supplementary Figure 2: Gene expression response to 2PE-ITC in *D. floralis*

Supplementary Figure 3: Gene expression response to 4MSOB-ITC in *D. radicum*

Supplementary Figure 4: Gene expression response to 4MSOB-ITC in *D. floralis*

**Supplementary Table 1.** Externally provided

**Supplementary Table 2.** Primer information of the mercapturic acid conjugation candidate genes.

| Gene | Name | Type | Forward primer | Reverse primer | Amplicon length | Primer reference |
| --- | --- | --- | --- | --- | --- | --- |
| GAPDH | Glyceraldehyde 3-phosphate dehydrogenase | Housekeeping gene | ACTGTCCATGCCACTACTGC | GGACACGGAAAGCCATACCA | 178bp | gene_36118 |
| EF-1α | Elongation factor 1 alpha | Housekeeping gene | GGGCATGTTGGATGAGGGTT | CGAGGCCCGTTATGAGGAAA | 139bp | gene_77018 |
| GST | Glutathione-*S*-transferase | Mercapturic acid pathway | AAATCTTCCTGCTGATCCGG | ACTGTTAATGAGTCGCCAGC | 108bp | gene_52573 |
| yGT | γ-glutamyltransferase | Mercapturic acid pathway | TGATTTTAGCACCACCAGCA | CCATCTTCACCGGCCAATTA | 127bp | gene_36580 |
| dipep | Dipeptidase | Mercapturic acid pathway | AGGGTGTTGGCTGATTTTCA | GCTTCTGCCTGGGATCTATT | 142bp | gene_14988 |
| NAcT | *N*-acetyltransferase | Mercapturic acid pathway | TCGAACGGGATGCGATATTT | GGTGCTCTGGGTTTACTTCA | 148bp | gene_11917 |
| CYP6A1 | Cytochrome P450 | Cytochrome P450 | ATGTTAGGTATGCGCACGAA | TAATATCACTGCCGCAGGTC | 123bp | gene_73068 |

#

**Supplementary Table 3**. Glucosinolates (in µM), isothiocyanates and their breakdown products (Blank corrected peak area) measured in gut extracts from *D. radicum* and *D. floralis* larvae incubated with 2-phenylethyl glucosinolate (2PE-GSL), 4-(methylsulfinyl)butyl glucosinolate (4MSOB-GSL), 2-phenylethyl isothiocyanate (2PE-ITC), or 4-(methylsulfinyl)butyl isothiocyanate (4MSOB-ITC). 2PE-amine: 2-phenylethylamine, 4MSOB-amine: 4-(methylsulfinyl)butylamine, 4MSOB-cysteine: 4-(methylsulfinyl)butyl cysteine, c4MSOB-cysteine: cyclic 4-(methylsulfinyl)butyl cysteine, n.d.: not detected

|  | *Delia radicum* | |  | *Delia floralis* | |  |
| --- | --- | --- | --- | --- | --- | --- |
| compound | Normal gut extracts with external GSL/ITC | Heated gut extracts with external GSL/ITC | Normal gut extracts without external GSL/ITC | Normal gut extracts with external GSL/ITC | Heated gut extracts with external GSL/ITC | Normal gut extracts without external GSL/ITC |
| 2PE-GSL [µM] | 37.21 | 36.56 | n.d. | 26.51 | 25.49 | n.d. |
|  | 33.15 | 36.57 | n.d. | 28.35 | 27.79 | n.d. |
|  | 35.24 | 36.59 | n.d. | 27.67 | 26.55 | n.d. |
| 4MSOB-GSL [µM] | 27.15 | 26.27 | n.d. | 26.42 | 34.03 | n.d. |
|  | 22.77 | 27.03 | n.d. | 19.41 | 21.45 | n.d. |
|  | 25.00 | 27.29 | n.d. | 18.66 | 26.01 | n.d. |
| 2PE-amine [peak area] | 5,899,240 | 7360 | n.d. | 6,809,240 | 6,849.56 | n.d. |
|  | 4,959,240 | 5220 | n.d. | 7,869,240 | - | n.d. |
|  | 4,199,240 | 7010 | n.d. | 6,059,250 | - | n.d. |
| 4MSOB-ITC [peak area] | 1,592,580 | 8,192,580 | n.d. | 2,502,580 | 5,692,580 | n.d. |
|  | 3,242,580 | 8,032,580 | n.d. | 4,432,580 | 5,952,580 | n.d. |
|  | 3,282,580 | 8,302,580 | n.d. | 1,902,580 | 6,732,580 | n.d. |
| 4MSOB-amine [peak area] | 3,044,440 | 35,340 | n.d. | 776,440 | 1,440 | n.d. |
|  | 1,824,440 | 51,640 | n.d. | 984,440 | 24,840 | n.d. |
|  | 3,634,440 | 64,440 | n.d. | 1,134,440 | 52,140 | n.d. |
| 4MSOB-cysteine [peak area] | 1,039,138 | 19,238 | n.d. | 1,269,138 | 140,138 | n.d. |
|  | 675,138 | 13,538 | n.d. | 1,289,138 | 148,138 | n.d. |
|  | 773,138 | 21,338 | n.d. | 1,729,138 | 87,938 | n.d. |
| c4MSOB-cysteine [peak area] | 24,984 | 624 | n.d. | 25,984 | 2,354 | n.d. |
|  | 21,384 | 254 | n.d. | 26,084 | 4,184 | n.d. |
|  | 36,084 | 744 | n.d. | 35,684 | 3,374 | n.d. |

**Supplementary Table 4.** One-way ANOVA of the isothiocyanate (ITC) breakdown experiment. Content of ITC and derived products in normal and heated gut extracts from *Delia radicum* or *D. floralis* larvae incubated with 4-(methylsulfinyl)butyl isothiocyanate (4MSOB-ITC). Each condition was presented by three replicates consisting of a pool of 10 larval guts. Two-way ANOVA of transcripts in *Delia radicum* or *D. floralis* larvae in the qPCR experiment. Larvae were treated with different ITCs (2-phenylethyl isothiocyanate (2PE-ITC) and 4MSOB-ITC) at different time points (6h, 24h, 48h), relative to untreated controls. Each condition and time point were represented by 4 till 5 replicates, depending on the survival of the larvae.

| **Experiment** | **compound** | **species** | **gene** | **condition** | **df** | **F-value** | **p-value** |
| --- | --- | --- | --- | --- | --- | --- | --- |
| ITC breakdown | 4MSOB-ITC | *D. radicum* | - | Heated gut extracts | 3 | 401.3 | < 0.001 |
|  |  |  |  | Normal gut extracts | 3 | 134.4 | < 0.001 |
|  |  | *D. floralis* | - | Heated gut extracts | 3 | 34.84 | < 0.001 |
|  |  |  |  | Normal gut extracts | 3 | 173.9 | < 0.001 |
| qPCR | 4MSOB-ITC | *D. radicum* | Glutathione-*S*-transferase | Time | 2 | 3.735 | 0.034 |
|  |  |  |  | Concentration | 2 | 1.651 | 0.206 |
|  |  |  |  | Time:concentration | 4 | 2.565 | 0.056 |
|  |  |  | γ-glutamyl-transferase | Time | 2 | 0.127 | 0.881 |
|  |  |  |  | Concentration | 2 | 3.910 | 0.030 |
|  |  |  |  | Time:concentration | 4 | 0.419 | 0.794 |
|  |  |  | Dipeptidae | Time | 2 | 2.992 | 0.064 |
|  |  |  |  | Concentration | 2 | 1.562 | 0.225 |
|  |  |  |  | Time:concentration | 4 | 0.940 | 0.453 |
|  |  |  | *N*-Acetyl-transferase | Time | 2 | 2.882 | 0.070 |
|  |  |  |  | Concentration | 2 | 1.307 | 0.284 |
|  |  |  |  | Time:concentration | 4 | 1.308 | 0.287 |
|  |  |  | CYP6A1 | Time | 2 | 1.364 | 0.269 |
|  |  |  |  | Concentration | 2 | 2.885 | 0.069 |
|  |  |  |  | Time:concentration | 4 | 0.885 | 0.482 |
|  |  | *D. floralis* | Glutathione-*S*-transferase | Time | 2 | 4.736 | 0.015 |
|  |  |  |  | Concentration | 2 | 5.705 | 0.007 |
|  |  |  |  | Time:concentration | 4 | 1.080 | 0.382 |
|  |  |  | γ-glutamyl-transferase | Time | 2 | 8.274 | 0.001 |
|  |  |  |  | Concentration | 2 | 0.340 | 0.714 |
|  |  |  |  | Time:concentration | 4 | 1.831 | 0.147 |
|  |  |  | Dipeptidae | Time | 2 | 12.860 | < 0.001 |
|  |  |  |  | Concentration | 2 | 2.790 | 0.076 |
|  |  |  |  | Time:concentration | 4 | 1.489 | 0.228 |
|  |  |  | *N*-Acetyl-transferase | Time | 1 | 12.236 | 0.002 |
|  |  |  |  | Concentration | 2 | 1.493 | 0.247 |
|  |  |  |  | Time:concentration | 2 | 3.793 | 0.038 |
|  | 2PE-ITC | *D. radicum* | Glutathione-*S*-transferase | Time | 2 | 14.817 | < 0.001 |
|  |  |  |  | Concentration | 2 | 7.382 | 0.002 |
|  |  |  |  | Time:concentration | 4 | 6.890 | < 0.001 |
|  |  |  | γ-glutamyl-transferase | Time | 2 | 9.820 | < 0.001 |
|  |  |  |  | Concentration | 2 | 1.600 | 0.2182 |
|  |  |  |  | Time:concentration | 4 | 4.847 | 0.004 |
|  |  |  | Dipeptidae | Time | 2 | 2.296 | 0.117 |
|  |  |  |  | Concentration | 2 | 13.669 | < 0.001 |
|  |  |  |  | Time:concentration | 4 | 1.460 | 0.238 |
|  |  |  | *N*-Acetyl-transferase | Time | 2 | 9.860 | < 0.001 |
|  |  |  |  | Concentration | 2 | 0.838 | 0.443 |
|  |  |  |  | Time:concentration | 4 | 2.653 | 0.052 |
|  |  |  | CYP6A1 | Time | 2 | 2.395 | 0.108 |
|  |  |  |  | Concentration | 2 | 8.541 | 0.001 |
|  |  |  |  | Time:concentration | 4 | 3.321 | 0.023 |
|  |  | *D. floralis* | Glutathione-*S*-transferase | Time | 1 | 0.379 | 0.546 |
|  |  |  |  | Concentration | 2 | 0.701 | 0.509 |
|  |  |  |  | Time:concentration | 2 | 0.190 | 0.829 |
|  |  |  | γ-glutamyl-transferase | Time | 1 | 0.144 | 0.708 |
|  |  |  |  | Concentration | 2 | 0.661 | 0.528 |
|  |  |  |  | Time:concentration | 2 | 0.942 | 0.408 |
|  |  |  | Dipeptidae | Time | 1 | 0.889 | 0.358 |
|  |  |  |  | Concentration | 2 | 5.470 | 0.014 |
|  |  |  |  | Time:concentration | 2 | 0.119 | 0.889 |
|  |  |  | *N*-Acetyl-transferase | Time | 1 | 1.743 | 0.203 |
|  |  |  |  | Concentration | 2 | 2.075 | 0.155 |
|  |  |  |  | Time:concentration | 2 | 0.238 | 0.791 |

**Supplement Table 5.** Female-male distribution resulting in *Delia radicum* and *D. floralis* adults when larvae fed on a semi-artificial diet containing 2-phenylethylamine (2PE-amine), 4-(methylsulfinyl)butylamine (4MSOB-amine), 4-(methylsulfinyl)butyl isothiocyanate (4MSOB-ITC) or pure diet. Female-male distribution was compared to a 1:1 ratio using a one-proportion z-test.

| **species** | **condition** | **females** | **males** | **p-value** |
| --- | --- | --- | --- | --- |
| *D. radicum* | control | 21 | 20 | 1 |
|  | 2PE-amine | 9 | 1 | 0.021 * |
|  | 4MSOB-amine | 17 | 4 | 0.007 ** |
|  | 4MSOB-ITC | 18 | 9 | 0.122 |
| *D. floralis* | control | 6 | 5 | 1 |
|  | 2PE-amine | 9 | 9 | 1 |
|  | 4MSOB-amine | 25 | 15 | 0.154 |
|  | 4MSOB-ITC | 14 | 12 | 0.845 |


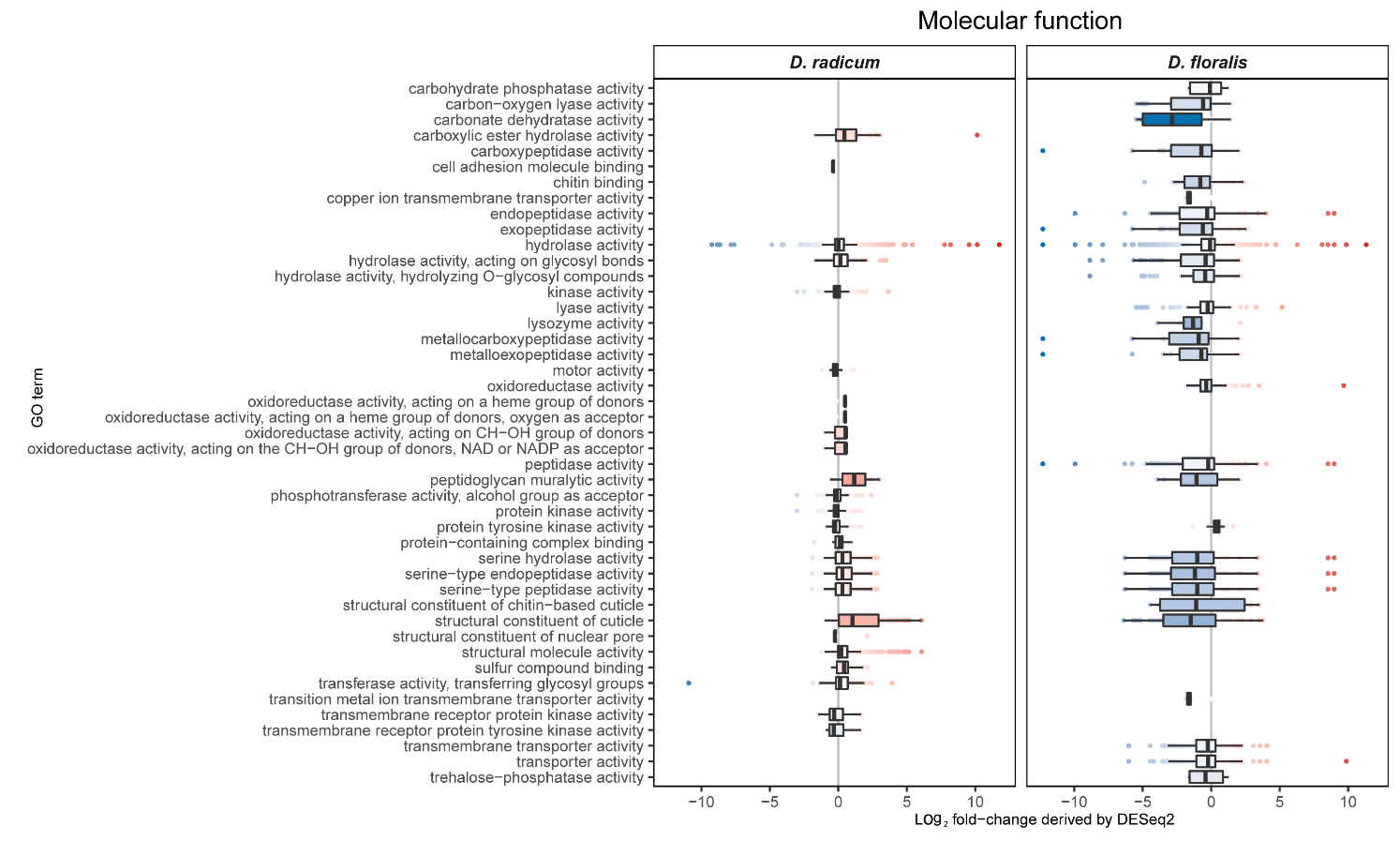
**Supplementary Figure 1.** Gene ontology analyses of molecular functions based on differently expressed GO terms in *Delia radicum* and *D. floralis* larvae exposed to 2-phenylethyl isothiocyanate (2PE- ITC) in their diet. Box plots show the distribution of Log_2_ fold-changes. Results are presented by medians (horizontal bar), interquartile ranges (IQRs; boxes), and data ranges (whiskers) excluding outliers (defined as > 1.5 x IQR). Rows (boxes) are labeled by GO terms. Only GO-terms that were significantly differentially observed between control and 2PE-ITC in a GO-enrichment analysis (p<0.05) are considered. Red colors: upregulated, blue colors: downregulated, intensity of color: strength of up- or downregulation.


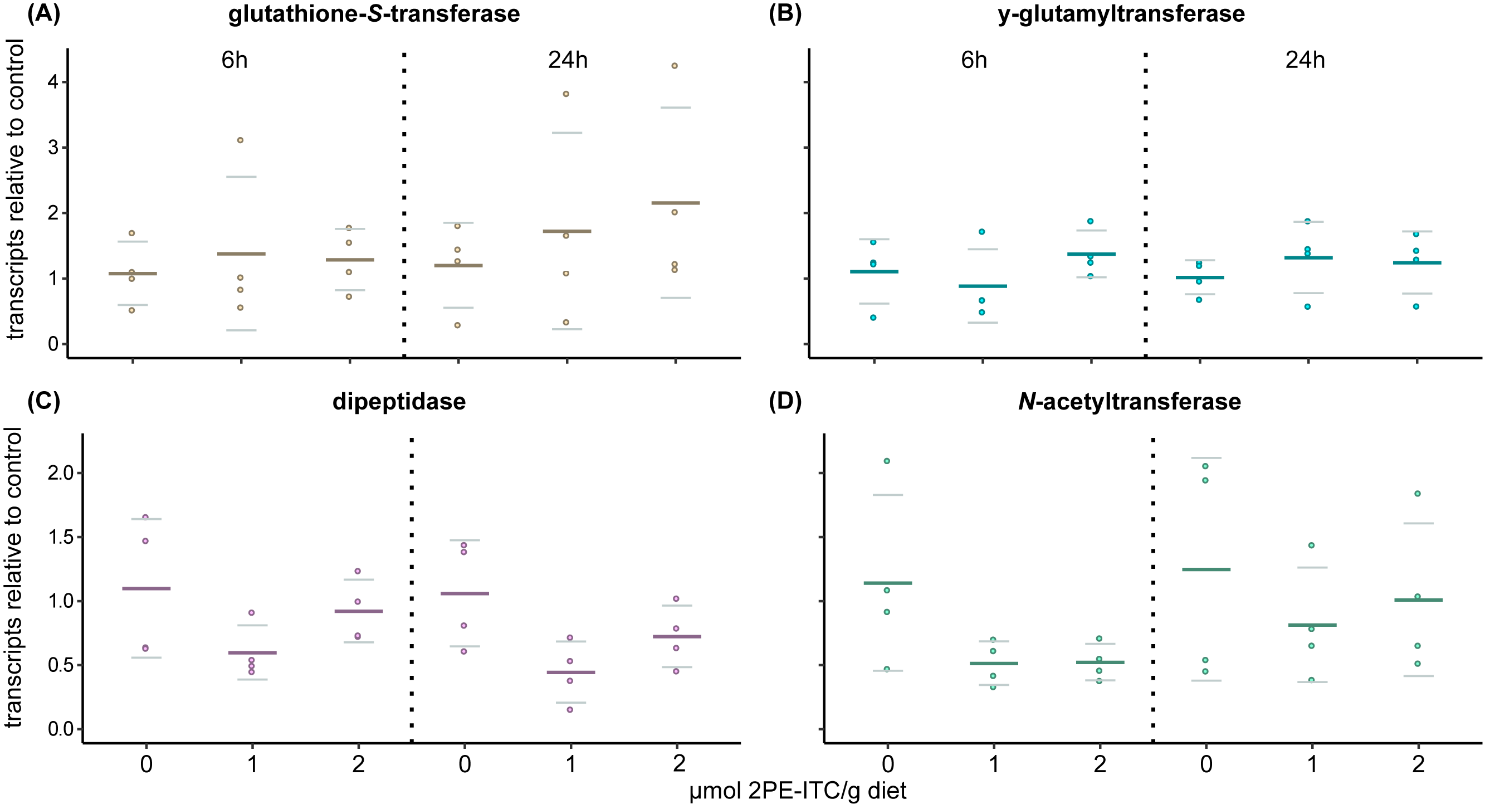
**Supplementary Figure 2.** Expression of selected candidate genes of the mercapturic acid pathway responding to 2-phenylethyl-isothiocyanates (2PE-ITC) in the diet of *D. floralis* larvae. Larvae were fed on 0, 1 or 2µmol/g diet of 2PE-ITC for 6h, 24h and 48h and the response of transcripts of a candidate gene coding for glutathione-*S*-transferase **(A)**, γ-glutamyltransferase **(B)**, dipeptidase **(C)** and *N*-acetyltransferase **(D)** compared relative to the control sample using 2^-ΔΔCt^. Mean values were present as dark lines, standard deviation as grey lines and individual values as dots.


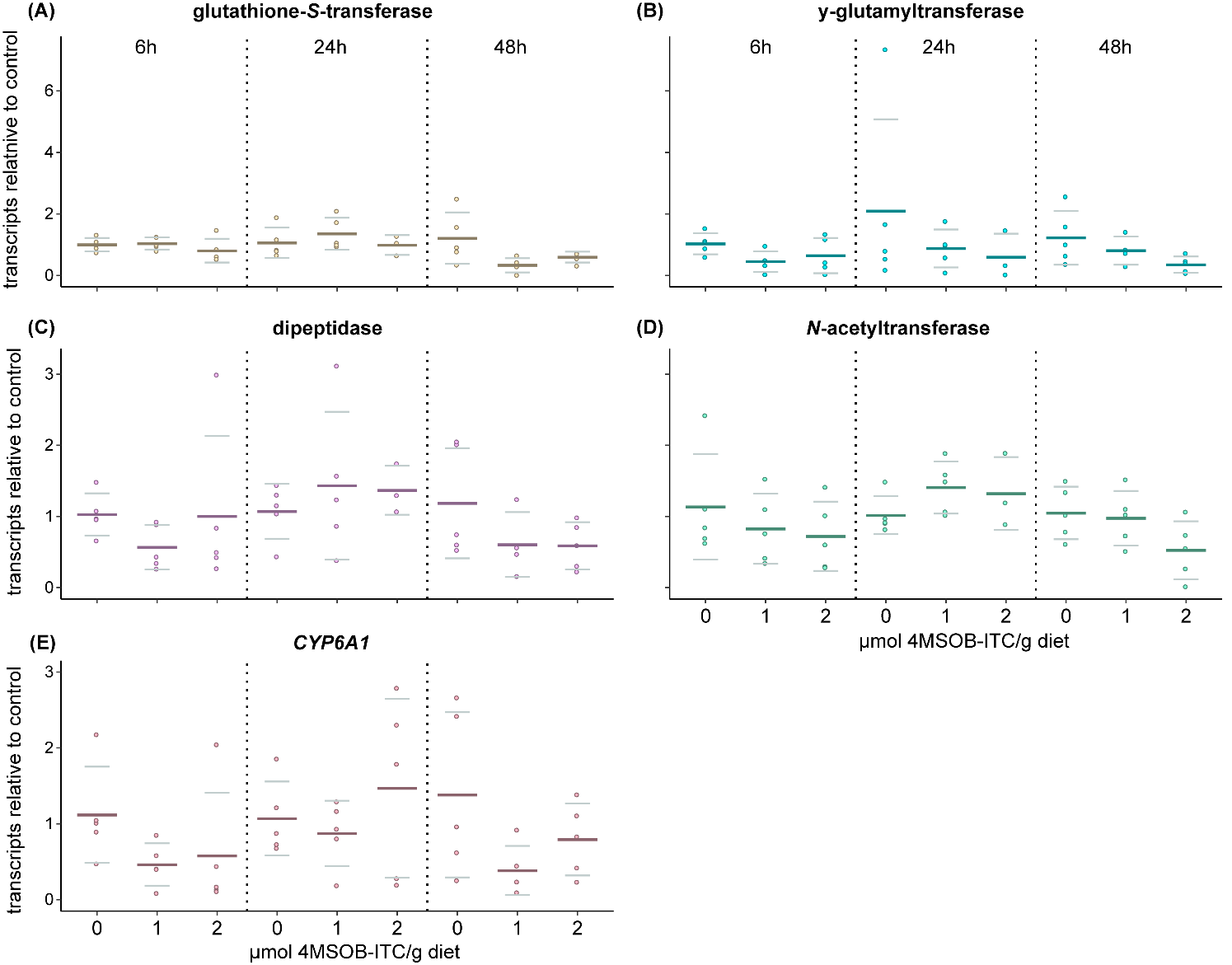


**Supplementary Figure 3.** Expression of selected candidate genes of the mercapturic acid pathway (A-D) and cytochrome P450 (*CYP6A1*, E) responding to 4-(methylsulfinyl)butyl isothiocyanate (4MSOB-ITC) in the diet of *D. radicum* larvae. Larvae were fed on 0, 1 or 2µmol/g diet 4MSOB-ITC for 6h, 24h and 48h and the response of transcripts of a candidate gene coding for glutathione-*S*-transferase (A), γ-glutamyltransferase (B), dipeptidase (C), *N*-acetyltransferase (D) and CYP6A1 (E) compared relative to the control sample using 2^-ΔΔCt^. Mean values were present as dark lines, standard deviation as grey lines and individual values as dots.


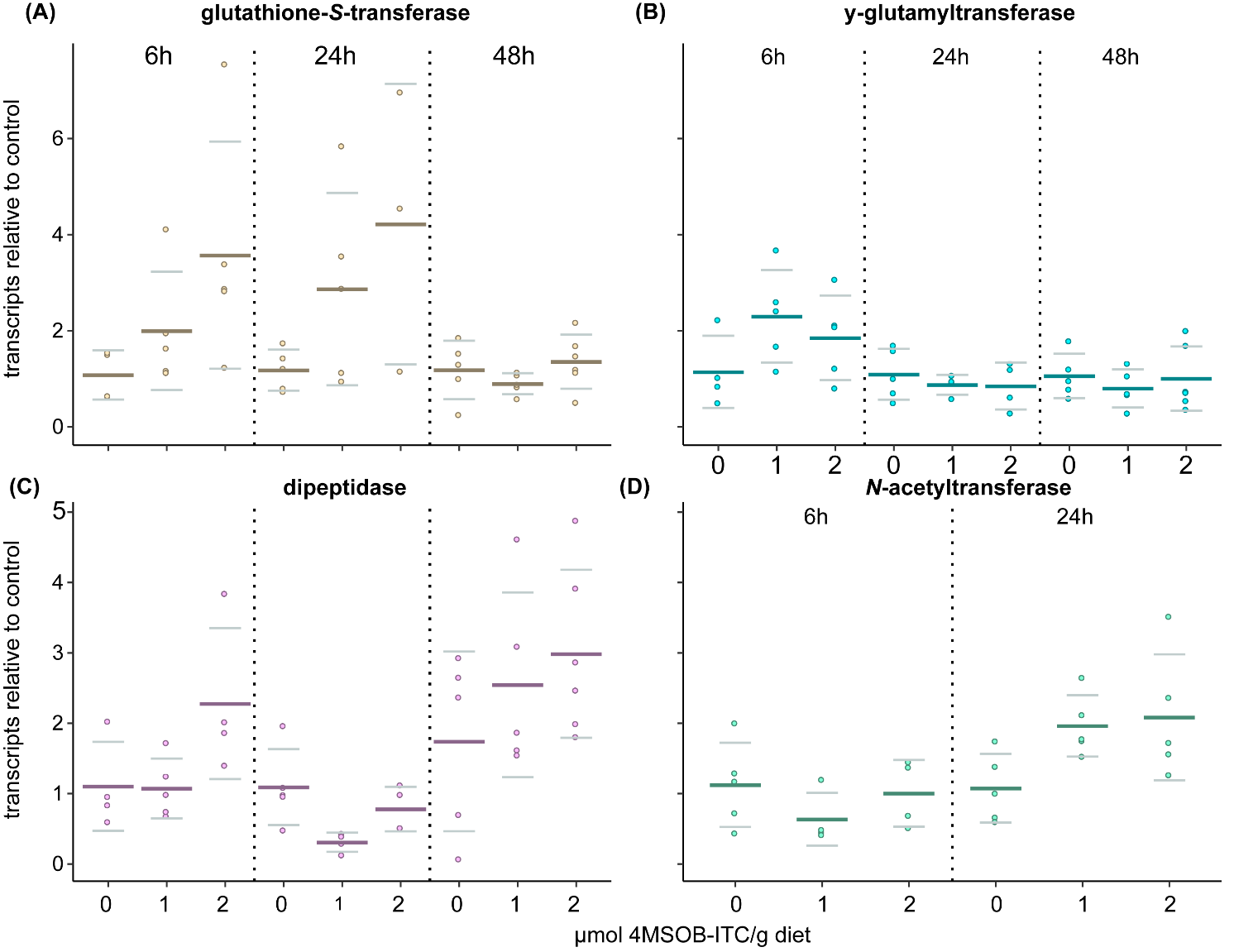


**Supplementary Figure 4.** Expression of selected candidate genes of the mercapturic acid pathway responding to 4-(methylsulfinyl)butyl isothiocyanates (4MSOB-ITC) in the diet of *D. floralis* larvae. Larvae were fed on 0, 1 or 2µmol/g diet of 4MSOB-ITC for 6h, 24h and 48h and the response of transcripts of a candidate gene coding for glutathione-*S*-transferase **(A)**, γ-glutamyltransferase **(B)**, dipeptidase **(C)** and *N*-acetyltransferase **(D)** compared relative to the control sample using 2^-ΔΔCt^. Mean values were present as dark lines, standard deviation as grey lines and individual values as dots.


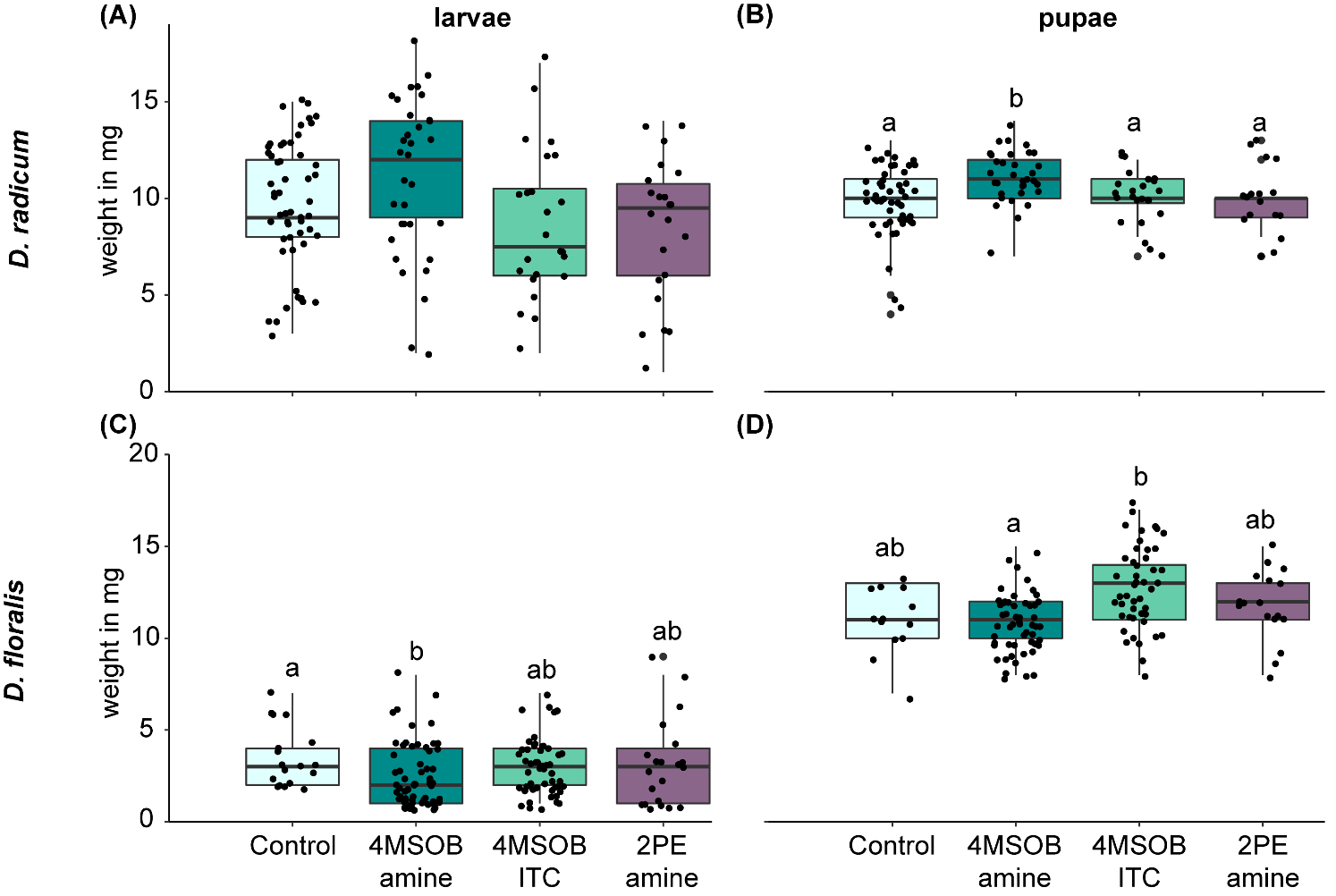


**Supplementary Figure 5.** Effects of isothiocyanates (ITC) and amines on larval and pupal weight in *Delia radicum* and *D. floralis*. Larvae were fed on a semi-artificial diet (control) or the same diet spiked with 2 µmol/g diet of 2-phenylethyl ITC (2PE-ITC), 2-phenylethylamine (2PE-amine), 4-(methylsulfinyl)butyl ITC (4MSOB-ITC), or 4-(methylsulfinyl)butylamine (4MSOB-amine). **(A)** Larval weight of *D. radicum,* **(B)** Pupal weight of *D. radicum,* **(C)** Larval weight of *D. floralis* and **(D)** Pupal weight of *D. radicum*. Different letters indicate significant differences between the treatments with a p-value < 0.05 performed with the Mann-Whitney test and a Bonferroni correction.


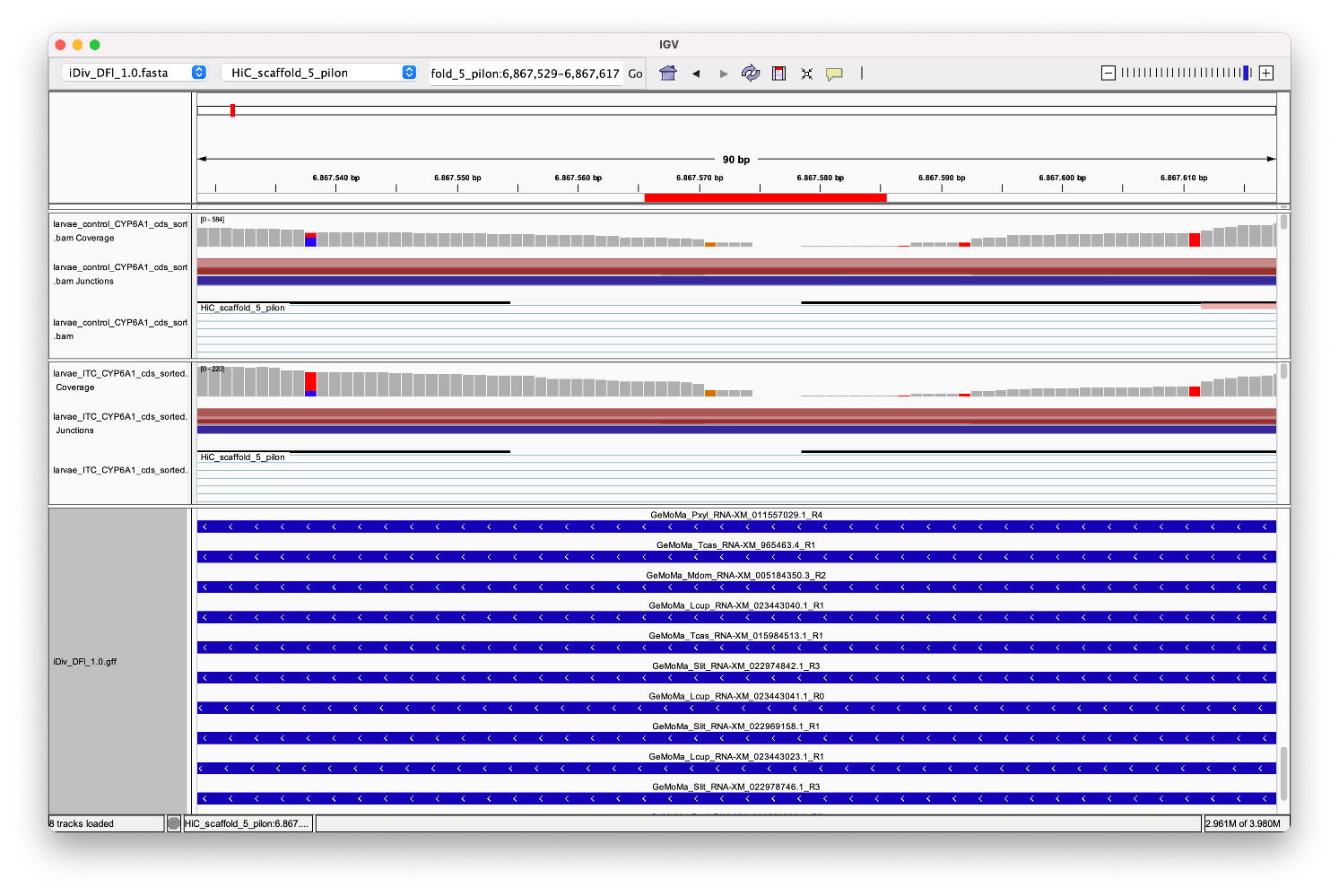


**Supplementary Figure 6.** Coverage of transcriptional *D. floralis* reads (grey bars) on a section of the *CYP6A1* gene region. Larvae were fed on a semi-artificial diet (control, 2^nd^ row) or the same diet spiked with 2 µmol/g diet of 2-phenylethyl ITC (ITC, 3^rd^ row). The first row presents the *CYP6A1* gene region including the amplified sequence selected for qPCR (red bar). No or only few reads mapped in the selected region which was not the case at the flank regions (left and right of the read bar)
